# The adaptor protein Grb2b is an essential modulator for lympho-venous sprout formation in the zebrafish trunk

**DOI:** 10.1007/s10456-021-09774-w

**Published:** 2021-03-07

**Authors:** Cristina Mauri, Andreas van Impel, Eirinn William Mackay, Stefan Schulte-Merker

**Affiliations:** 1 Institute for Cardiovascular Organogenesis and Regeneration, Faculty of Medicine, WWU Münster, Münster, Germany; 2 Cells-in-Motion Cluster of Excellence, WWU Münster, Münster, Germany; 3 Hubrecht Institute - KNAW & UMC Utrecht, 3584 CT Utrecht, The Netherlands

**Keywords:** Lymphangiogenesis, Angiogenesis, Development, Zebrafish, Vasculature, VEGFR3

## Abstract

Vegfc/Vegfr3 signaling is critical for lymphangiogenesis, the sprouting of lymphatic vessels. In zebrafish, cells sprouting from the posterior cardinal vein can either form lymphatic precursor cells or contribute to intersegmental vein formation. Both, the Vegfc-dependent differential induction of Prox1a in sprouting cells as well as a Notch-mediated pre-pattern within intersegmental vessels have been associated with the regulation of secondary sprout behavior. However, how exactly a differential lymphatic versus venous sprout cell behavior is achieved is not fully understood. Here, we characterize a zebrafish mutant in the adaptor protein Grb2b, and demonstrate through genetic interaction studies that Grb2b acts within the Vegfr3 pathway. Mutant embryos exhibit phenotypes that are consistent with reduced Vegfr3 signaling outputs prior to the sprouting of endothelial cells from the vein. During secondary sprouting stages, loss of *grb2b* leads to defective cell behaviors resulting in a loss of parachordal lymphangioblasts, while only partially affecting the number of intersegmental veins. A second GRB2 zebrafish ortholog, *grb2a*, contributes to the development of lymphatic structures in the meninges and in the head, but not in the trunk. Our results illustrate an essential role of Grb2b in vivo for cell migration to the horizontal myoseptum and for the correct formation of the lymphatic vasculature, while being less critically required in intersegmental vein formation. Thus, there appear to be higher requirements for Grb2b and therefore Vegfr3 downstream signaling levels in lymphatic versus vein precursor-generating sprouts.

**Supplementary Information:**

The online version contains supplementary material available at 10.1007/s10456-021-09774-w.

## Introduction

The lymphatic vasculature is a vital organ system that covers different functions in the vertebrate body, such as trafficking of immune cells, maintenance of fluid homeostasis and absorption of dietary fat. Malfunctioning of lymphatic vessels can cause pathological conditions, such as lymphedema or inflammation [1, 2]. Lymphangiogenesis is largely dependent on the vascular endothelial growth factor C (VEGFC)/vascular endothelial growth factor receptor 3 (VEGFR3, also termed FLT4) signaling pathway. Briefly, the secreted ligand VEGFC is produced as a pre-pro-protein and it is activated by proteolytical cleavage at both C and N terminus. Furin/PC5, collagen and calcium-binding EGF domain 1 (CCBE1) and two different members of the A disintegrin and metalloprotease with thrombospondin motifs (ADAMTS) protein family, ADAMTS3 and ADAMTS14, are central players in this cleavage process [3–6]. Once proteolytically activated, a VEGFC dimer binds with high affinity to its receptor VEGFR3, triggering intracellular downstream signaling events in endothelial cells. As functional or structural impairments of the lymphatic system result in pathological conditions in humans, understanding how the lymphatic vasculature develops and which genes are essential for its functionality is extremely important. At the cellular level, the system is best understood in mice [7] and zebrafish (*Danio rerio*) [8]. In zebrafish, at around 32–36 h post-fertilization (hpf), cells sprout from the posterior cardinal vein (PCV) and exhibit either one of two different behaviors: Half of the cells will connect to an arterial intersegmental vessel (aISV), remodeling it into a venous intersegmental vessel (vISV). Consequently, zebrafish embryos develop arteries and veins in a 1:1 ratio within the trunk [9]. The other half migrates to the horizontal myoseptum (HM) to constitute a pool of lymphatic precursor cells, so-called parachordal lymphangioblasts (PLs). Later, these cells migrate either ventrally or dorsally forming the thoracic duct (TD) and the dorsal longitudinal lymphatic vessel (DLLV), respectively, the two major lymphatic vessels in the trunk which are completed at 5 days post-fertilization (dpf) [1, 8].

The distinguishing features between venous and lymphatic sprouts, which both depend on active Vegfc signaling [4, 6, 10], have been a matter of contention for considerable time [11–13]. More recently, the Vegfc-dependent upregulation of Prox1a expression in 65% of secondary sprouts has been associated with a lymphatic cell behavior, suggesting that the decision whether to generate an intersegmental vein or a lymphatic precursor cell would be made at the level of the nascent secondary sprouts [13]. A different study, however, indicated that the balanced number of intersegmental arteries and veins is determined by a Notch-mediated pre-patterning within intersegmental vessels and by flow-dependent endothelial cell migration [14]. Primary ISVs would be pre-specified to become either arteries or veins already before the onset of secondary sprouting, suggesting that the decision whether a secondary sprout shows a ‘venous’ or ‘lymphatic’ cell behavior at a given position in the embryo is governed by the Notch levels in the nearby ISV [14]. Despite the apparent tight interdependency of artery-vein balance and lymphatic development, the molecular pathways controlling and regulating these different cell fate decisions have not been fully elucidated yet.

The importance of the Vegfc/Vegfr3 signaling pathway during secondary sprouting is evidenced by various mutant scenarios [4, 6, 10, 15]. Mutants have been analyzed for genes/proteins that affect Vegfc processing, and various allelic variations of Vegfr3 have been analyzed [4, 10, 16], but it is less clear through which specific downstream signaling components Vegfr3 exerts its effect. In principle, two main effector branches have been considered and analyzed in vitro, namely the serine/threonine kinases AKT and ERK/MAPK. Mutants for these downstream effector genes have thus far not been investigated in vivo. In the present study, we analyze the role of growth factor receptor-bound protein 2b (Grb2b) during lymphatic development in the zebrafish and its involvement in lympho-venous sprout formation in the trunk. GRB2 constitutes an adaptor protein that acts downstream not only of VEGFR3, but of different tyrosine kinase receptors [17]. It contains two Src homology 3 (SH3) and one Src homology 2 (SH2) domain [17]. The SH2 domain binds to phosphorylated tyrosine kinase receptors [17], whereas the SH3 domains are both needed for a stable interaction with proline-rich proteins, such as Sos [18, 19]. Here, we demonstrate that this adaptor protein, which has been shown to bind to VEGFR3 in vitro [20], is essential for normal endothelial cell migration in the zebrafish trunk and that it has differential effects on venous versus lymphatic endothelial cell behavior.

## Materials and methods

### Zebrafish husbandry and strains

Zebrafish (*Danio rerio*) strains were maintained under standard husbandry conditions, according to FELASA recommendations [21]. Animal experiments have been performed according to guidelines of the animal ethics committees at the University of Münster, Germany. Embryonic developmental stages were determined as previously described [22]. Transgenic lines used in this work are: *Tg(flt4:mCitrine)*^*hu7135*^ [11], *Tg(flt1*^*enh*^*:tdTomato)*^*hu5333*^ [23], *Tg(fli1a:eGFP)*^*y1*^ [24], *Tg(fli1a:nEGFP)*^*y7*^ [25], *Tg(shh:vegfc-IRES-mTurquoise)*^*hu10933*^ [4], *Tg(flt4:Gal4FF)*^*hu9236*^ [11]*, Tg(UAS:GFP)*^*nkuasgfp1a*^ [26].

### Transgenesis

For the endothelial-specific rescue, the *Tg(UAS:grb2b-P2A-RFP)*^*mu406*^ was generated. Briefly, a 5xUAS:RFP-polyA cassette was inserted into the miniTol2 vector via a NotI restriction site. Subsequently, a zebrafish codon-optimized P2A sequence with a 5′ flanking KpnI restriction site was added directly upstream of the RFP coding sequence. In an additional step, the *grb2b* cDNA was inserted into the vector via the KpnI restriction site, using NEBuilder (New England BioLabs). 25 pg of the final construct together with 25 pg of Tol2 mRNA were injected into one-cell-stage embryos from an outcross of *tabula rasa* carriers with *flt4:Gal4FF; UAS:GFP* transgenic fish.

### Genome editing

CRISPR-mediated genome editing for the generation of *grb2b* and *grb2a* mutants was performed as described [27]. The respective sgRNA target sites in *grb2b* (exon 2) was 5′- GATGAGCTGAGTTTTAAACG-3′, and in *grb2a* (exon 4) 5′-TGGGAAGATTCCCCGTGCAAA-3’.

### Morpholino (MO) injections

*vegfr3* ATG MO (5′-CTCTTCATTTCCAGGTTTCAAGTCC-3′) was injected at 0.15 ng/embryo, the *plcγ-1* splicing MO (5′- ATTAGCATAGGGAACTTACTTTCG-3′) at 10 ng/embryo, and the *dll4* splicing MO (5′-TGATCTCTGATTGCTTACGTTCTTC-3) at 4 ng/embryo.

### Genotyping

*grb2b* and *grb2a* were genotyped by KASPar. Primers used for *tabula rasa*: forward wt: 5′-AGAACTAAACGGCAAAGACGGTTTCAt-3′, forward mut: 5′-AGAACTAAACGGCAAAGACGGTTTCAa-3′; common reverse: 5′-GGTTGTGGTGGCTGAAGTTAAGCATC-3′. Primers used for *grb2b*^*mu404*^: forward wt: 5′-ACTGCAGATGATGAGCTGAGTTTTAa-3′, forward mut: 5′-ACTGCAGATGATGAGCTGAGTTTTAc-3′, common reverse: 5′-AACAAACATTAAATAACCATGACTTACCTTCA-3′. Primers used for *grb2a*^*mu405*^: forward wt: 5′-TGGTTTTATGGGAAGATTCCCCGTGc-3′, forward mut: 5′-TGGTTTTATGGGAAGATTCCCCGTGg-3′, common reverse: 5′-AAGAAAAGCTCCATCGTGCCTCTGTT-3′. *shh:zfvegfc* was genotyped by PCR: forward: 5′-GCAGTTGCGTTCAGCGGGTAGTGT-3′, reverse: 5′-GCTGATGTATGAAGTGCTGATGTT-3’.

### Antibodies

The following antibodies and reagents were used: Prox1 rabbit mAb (1:500, AngioBio Co #11-002), phospho-ERK1/2 antibody XP rabbit mAb (1:250, Cell signaling #4370), anti-GFP chicken polyclonal (1:400, ab13970), goat α-rabbit IgG-HRP (1:1000, Life Technologies), Tyramide-FITC/Cy3 (1:50, NEL744001KT, Perkin Elmer), Alexa-488 goat anti-chicken (1:200, Invitrogen, A11039).

### Immunohistochemistry

Embryos at 32hpf from a *grb2b*^*mu404*^ in-cross were fixed overnight and stained with either α-pErk and α-GFP, or with α-Prox1 and α-GFP according to a previously described protocol [4]. For Prox1 staining, the following modifications were used: after acetone treatment, embryos were treated with Proteinase K for 20 min at 37 °C. The embryos were imaged (lateral views) and pERK/Prox1 positive cells were quantified in the posterior cardinal vein by scoring co-expression of *flt4:mCitrine* detected by α-GFP in green and α-pERK/α-Prox1 in red across an area of 9 somites in the trunk.

### In situ hybridizations

Anti-sense RNA probes for *grb2b* and *grb2a* were generated by PCR from cDNA. Several different probes were tested for each gene which yielded similar staining results. Primers used were: grb2b_Ex2-4 For: 5′-ATGGAGGCCATTGCCAAGTATGA-3′; grb2b_Ex2-4 (T3 promoter) Rev: 5′-cattaaccctcactaaagggaaGCTGAACATCATTACCAAACTTGACAGAC-3′; grb2b_5′UTR For: 5′-AAGCGTGGATTCTGCGTTCA-3′; grb2b_5′UTR (T3 promoter) Rev: 5′-cattaaccctcactaaagggaaATTCAGCCAAACGGACACCA; grb2b_3′UTR For: 5′-CTGTGAAATCCAAAGCAGCA-3′; grb2b_3′UTR (T3 promoter) Rev: 5′-cattaaccctcactaaagggaaAAAAGAAGCCAAAAGCAGCA-3′; and grb2a_Ex2-4 For: 5′-ATGGAGGCAATAGCTAAATATGACTTCAAAG-3′; grb2a_Ex2-4 (T3 promoter) Rev: 5′-cattaaccctcactaaagggaaTTCCAAATTTAACAGAGAGCGAGAAGTCT-3′; grb2a_Ex5-6 For: 5′-AAAGTTTTACGGGACGGAGCTGG-3′; grb2a_Ex5-6 (T3 promoter) Rev: 5′-cattaaccctcactaaagggaaGTTACATGTTTTGATTGACAGGTGTGACA-3′; grb2a_5′UTR For: TCACTGCGAGACTACAAGGC; grb2a_5′UTR (T3 promoter) Rev: cattaaccctcactaaagggaaTCTTTGTCTGCTTGCCGTCA; grb2a_3′UTR For: CTGCATAAAGCACCTGTGGA; grb2a_3′UTR (T3 promoter) Rev: cattaaccctcactaaagggaaAGCTCCATCAAAAGCAAAGC. Probes were transcribed with T3 RNA polymerase and hybridizations were carried out on TL embryos as described before [28].

## Results

### Identification of a zebrafish *grb2b* mutant allele: the *tabula rasa* phenotype

**Fig. 1:**
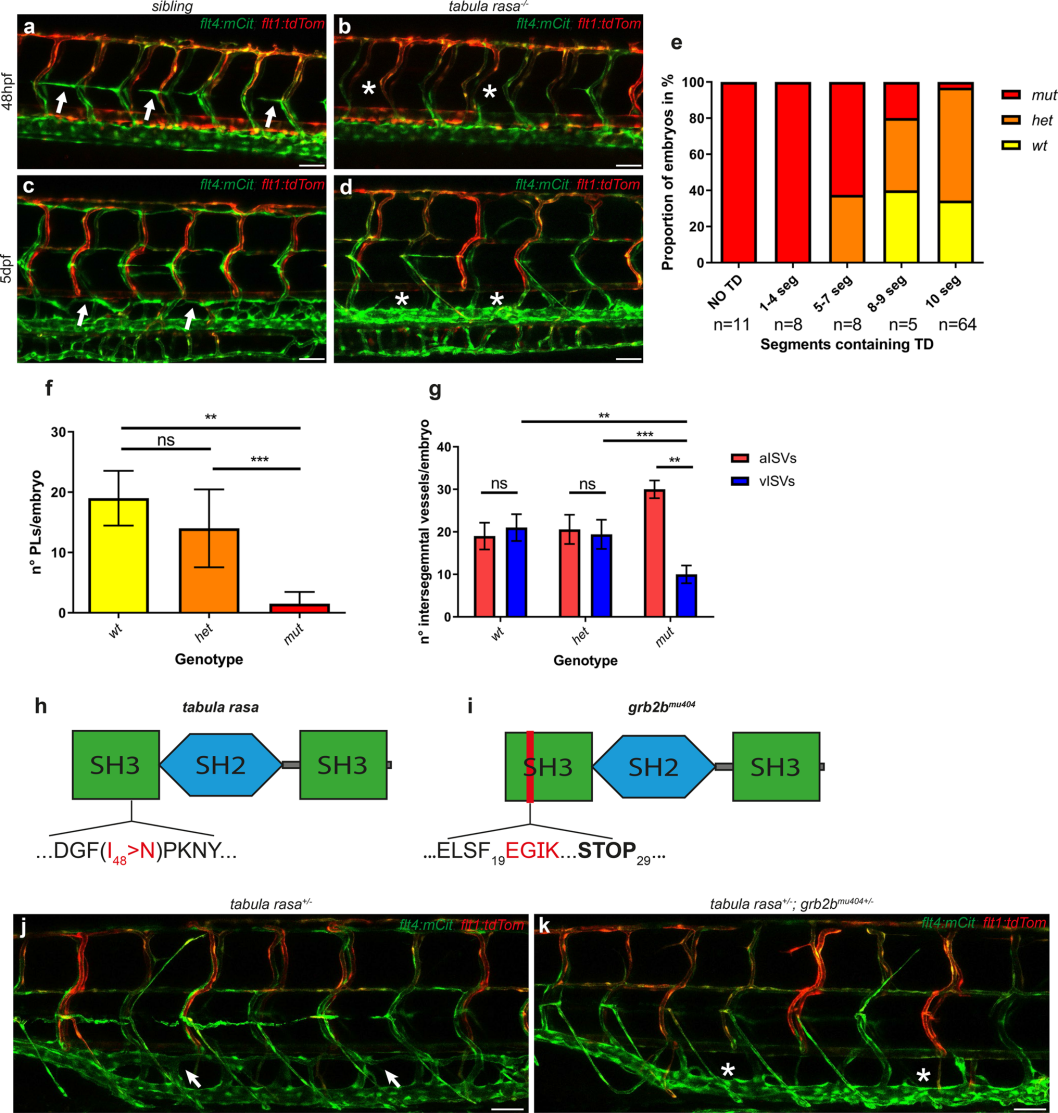
Mutations in the *grb2b* gene interfere with lymphatic development. **a**–**d** Confocal projections of the trunk region in siblings (**a**, **c**) and *tabula rasa* mutants (**b**, **d**) at 48hpf (**a**, **b**) and at 5dpf (**c**, **d**) with *flt4:mCitrine* highlighting venous and lymphatic structures in green and the *flt1:tdTomato* transgene showing arterial endothelial cells in red. Arrows indicate the presence of PLs at the HM (**a**) and of a fully developed TD (**c**) in siblings, whereas asterisks indicate the lack of PLs (**b**) and of TD fragments (**d**) in mutants. **e** Quantification of TD-containing segments scored over the length of 10 consecutive somites at 5dpf. Most *tabula rasa* mutants lack the whole TD or they form only few fragments. *wt*: *n* = 24; *het*: *n* = 45; *mut*: *n* = 27. **f** Quantification of the number of PLs per embryo at 48hpf shows a significant decrease in the number of PLs in *tabula rasa* mutants compared to both wild types and heterozygotes. *wt*: *n* = 4; *het*: *n* = 12; *mut*: *n* = 6. ** Between *wt* and *mut*: *P* value 0.0095 (Mann–Whitney). ***Between *het* and *mut*: *P* value 0.003 (Mann–Whitney). **g** Quantification of the number of aISVs and vISVs in 20 consecutive segments (bilateral) at 48hpf in the zebrafish trunk. *tabula rasa* mutants display significantly higher numbers of arteries than veins. *wt* *n* = 4; *het*: *n* = 12; *mut*: *n* = 6. **Between vISVs *wt* and vISVs *mut*: *P* value = 0.0048 (Mann–Whitney). ***Between vISVs *het* and vISVs *mut*: *P* value  = 0.0001 (Mann Whitney). ** Between aISVs *mut* and vISVs *mut*: *P* value 0.0022 (Mann–Whitney). **h**, **i** Schematic representations of the Grb2b protein containing two SH3 domains and one SH2 domain. **h** I_48_ > N indicates the point mutation in the *tabula rasa* mutant: the isoleucine at position 48 is replaced by an asparagine. **i** A 2 bp deletion in the *grb2b*^*mu404*^ allele is predicted to cause a frame-shift after amino acid 19, within the first SH3 domain of the protein. **j**, **k** Confocal projections showing the lack of complementation in *tabula rasa* and *grb2b*^*mu404*^ trans-heterozygous embryos. **j** *tabula rasa* heterozygous embryos have a full TD, as indicated by arrows. **k** *tabula rasa*^+/−^; *grb2b*^*mu404*+/−^ trans-heterozygous embryos lack the TD (indicated by asterisks). *flt4:mCitrine* is shown in green and *flt1:tdTomato* in red. *SH2* Src homology domain 2, *SH3* Src homology domain 3, *aISVs* arterial intersegmental vessels, *vISVs* venous intersegmental vessels, *PL* parachordal lymphangioblast, *HM* horizontal myoseptum, *TD* thoracic duct, *ns* not significant. Scale bars: 50 µm. Data in **f**, **g** are mean ± sd

In a forward genetic screen for mutations affecting the formation of the lymphatic vasculature in the zebrafish trunk, the *tabula rasa* mutant was isolated, in which the formation of the lymphatic vasculature was impaired, while exhibiting no overt phenotypes in other tissues at 5dpf. Embryos were scored for the presence of a TD in 10 consecutive segments within the trunk (above the yolk extension) at 5dpf. Phenotypes were divided into five categories, containing either TD fragments within 0, 1–4, 5–7, 8–9, or 10 segments. Most *tabula rasa* mutant embryos did not develop a TD at all, or showed only few fragments (in 1–4 segments). In only a small portion, an almost complete (in 8–9 segments) or a complete (in 10 segments) TD was present (Fig. 1c–e). Analysis of PLs at the HM at 48hpf revealed that the number of lymphatic precursor cells was strongly reduced in mutant embryos (Fig. 1a, b, f), whereas the aISVs/vISVs ratio was increased (Fig. 1g). Although the number of vISVs was significantly decreased compared to siblings, mutant embryos still exhibited on average 10 veins per embryo at 48hpf.

Genetic mapping based on whole-genome sequencing data from pooled mutant and sibling DNA [29] linked the causative mutation to chromosome 3 (Supplementary Fig. S1a). Subsequent analysis of the genomic candidate region revealed a missense mutation in the coding region of *grb2b* (Supplementary Fig. S1b), leading to an amino acid exchange at position 48 (exon 3) of the Grb2b protein (Fig. 1h). The amino acid exchange affects a highly conserved isoleucine within the first SH3 domain of the protein [30]. In order to show that the identified mutation within *grb2b* was causative for the lymphatic defects, *grb2b* mRNA was injected into embryos from a *tabula rasa* in-cross and embryos were analyzed at 5dpf. After injection of wild-type *grb2b* mRNA, the majority of mutant embryos indeed developed a TD (Supplementary Fig. S2a, b). In addition, we generated an independent *grb2b* allele, employing the CRISPR/Cas9 system. This allele, *grb2b*^*mu404*^, harbors a 2 bp deletion at the beginning of exon 2, resulting in a predicted premature stop codon at amino acid position 29 (Fig. 1i). Complementation assays between *grb2b*^*mu404*^ and *tabula rasa* confirmed that *tabula rasa* represents a *grb2b* allele, as trans-heterozygous embryos failed to form lymphatic structures in the trunk at 5dpf (Fig. 1j, k). Further analysis of *grb2b*^*mu404*^ mutant embryos demonstrated that this early stop allele completely recapitulated the *tabula rasa* mutant phenotype, causing equally strong defects in lymphatic development and in the ratio of arterial and venous ISVs (Supplementary Fig. S3a–c). We therefore conclude that *tabula rasa* represents a loss-of-function allele of the *grb2b* gene that impairs lymphatic development in the zebrafish trunk.

### Endothelial-specific expression of Grb2b rescues the *tabula rasa* lymphatic phenotype

**Fig. 2:**
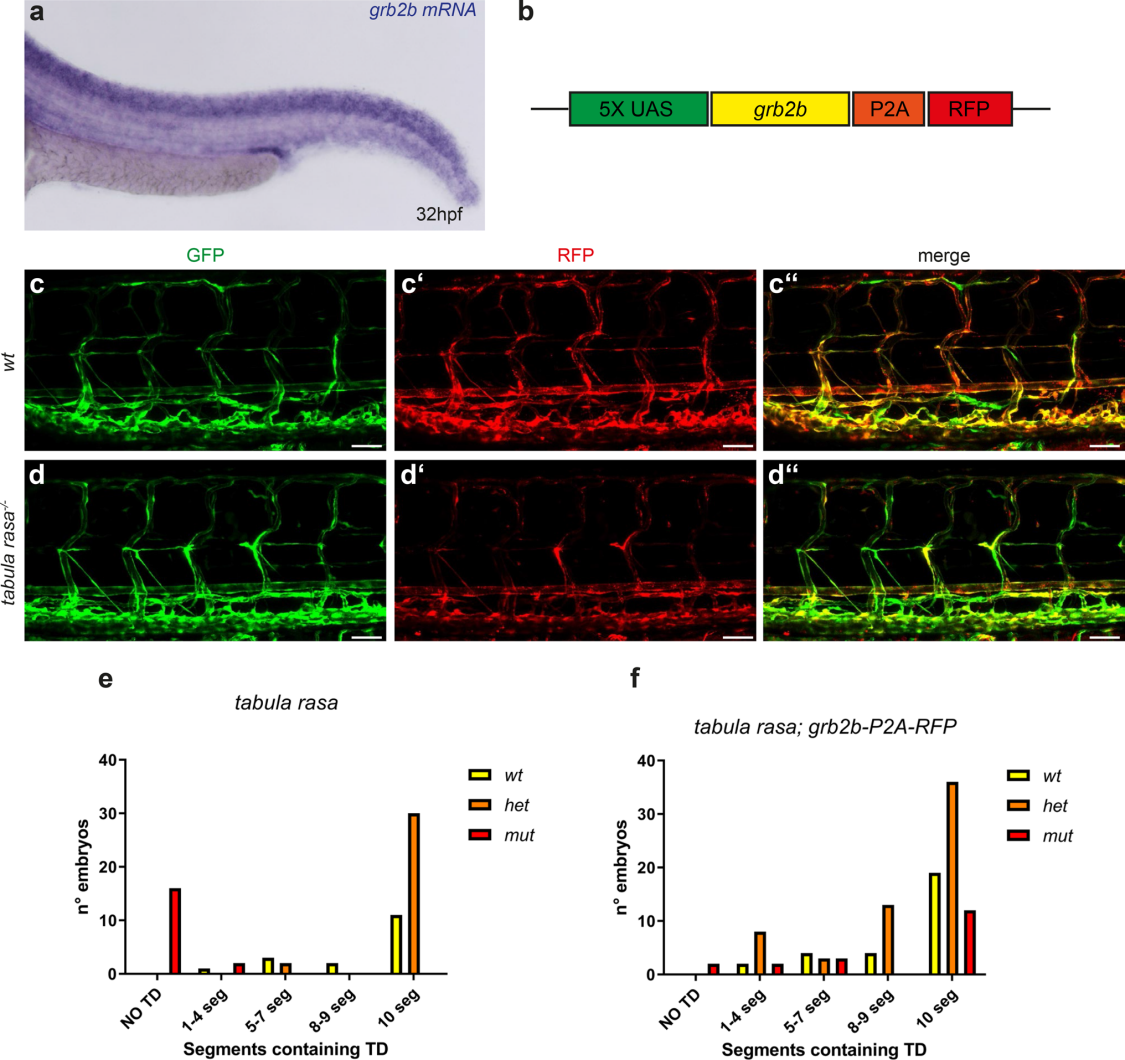
Endothelial-specific expression of *grb2b* is sufficient for normal lymphatic development. **a** In situ hybridization of *grb2b* shows expression in the majority of tissues in zebrafish embryos at 32hpf. **b** Rescue construct containing a 5xUAS element upstream of the *grb2b* cDNA which was fused with an RFP cassette via a P2A self-cleaving peptide. **c**–**d**ʺ Confocal projections of transgenic *flt4:Gal4; UAS:GFP*; *UAS:grb2b-P2A-RFP* embryos from a *tabula rasa* in-cross at 5dpf. A *tabula rasa* wild-type embryo is shown in **c**–**c**ʺ and a homozygous mutant in **d**–**d**ʺ. **e**, **f** Quantification of trunk segments containing TD (scored over the length of 10 somites) in 5dpf embryos from a *tabula rasa*^+/−^; *flt4:Gal4*; *UAS:GFP*; *grb2b-P2A-RFP* in-cross. **e** GFP^+^ RFP^−^ embryos (i.e., not containing the rescue construct) served as a control. *wt*: *n* = 17; *het*: *n* = 32; *mut*: *n* = 18*.* **f** The mutant phenotype is rescued in embryos expressing the rescue construct (GFP^+^ and RFP^+^). *wt*: *n* = 29; *het*: *n* = 60; *mut*: *n* = 19. *TD* thoracic duct, *wt* wild type; *het* heterozygous; *mut* mutant for *tabula rasa.* Scale bars: 50 µm

In order to analyze in which tissue *grb2b* is expressed, in situ hybridization was performed on 32hpf old embryos, revealing *grb2b* expression in the majority of tissues during venous sprouting stages, with increased expression levels in the central nervous system and part of the pronephros (Fig. 2a, Supplementary Fig. S4a–d). We therefore wanted to assess in which cells/tissues Grb2b activity is required for normal lymphatic development to occur and performed tissue-specific rescue experiments. To this end, we generated a construct containing the wild-type *grb2b* cDNA under control of a 5xUAS element. In order to highlight cells expressing the construct, a self-cleaving P2A peptide [31] followed by an RFP reporter cassette was added to the construct (Fig. 2b). We established a stable *Tg(UAS:grb2b-P2A-RFP)* transgenic line in a *flt4:Gal4FF*; *UAS:GFP*; *tabula rasa*^+/−^ background, which enabled us to drive the expression of Grb2b specifically in endothelial cells (as shown by RFP expression) (Fig. 2c-dʺ). While *tabula rasa* mutants lacking the rescue construct (GFP^+^, RFP^−^) showed a strong impairment of TD formation (88% of *tabula rasa* mutants completely lacked a TD), mutants expressing the construct (GFP^+^, RFP^+^) displayed a markedly milder phenotype: the majority of embryos (63%) developed a full TD, and only in a minority (37%) the TD was present in fewer segments or not formed at all (Fig. 2e, f). Hence, functional Grb2b within endothelial cells is sufficient to rescue the *tabula rasa* lymphatic phenotype and to enable normal lymphatic development.

### *grb2a* is dispensable for lymphatic formation in the zebrafish trunk

**Fig. 3:**
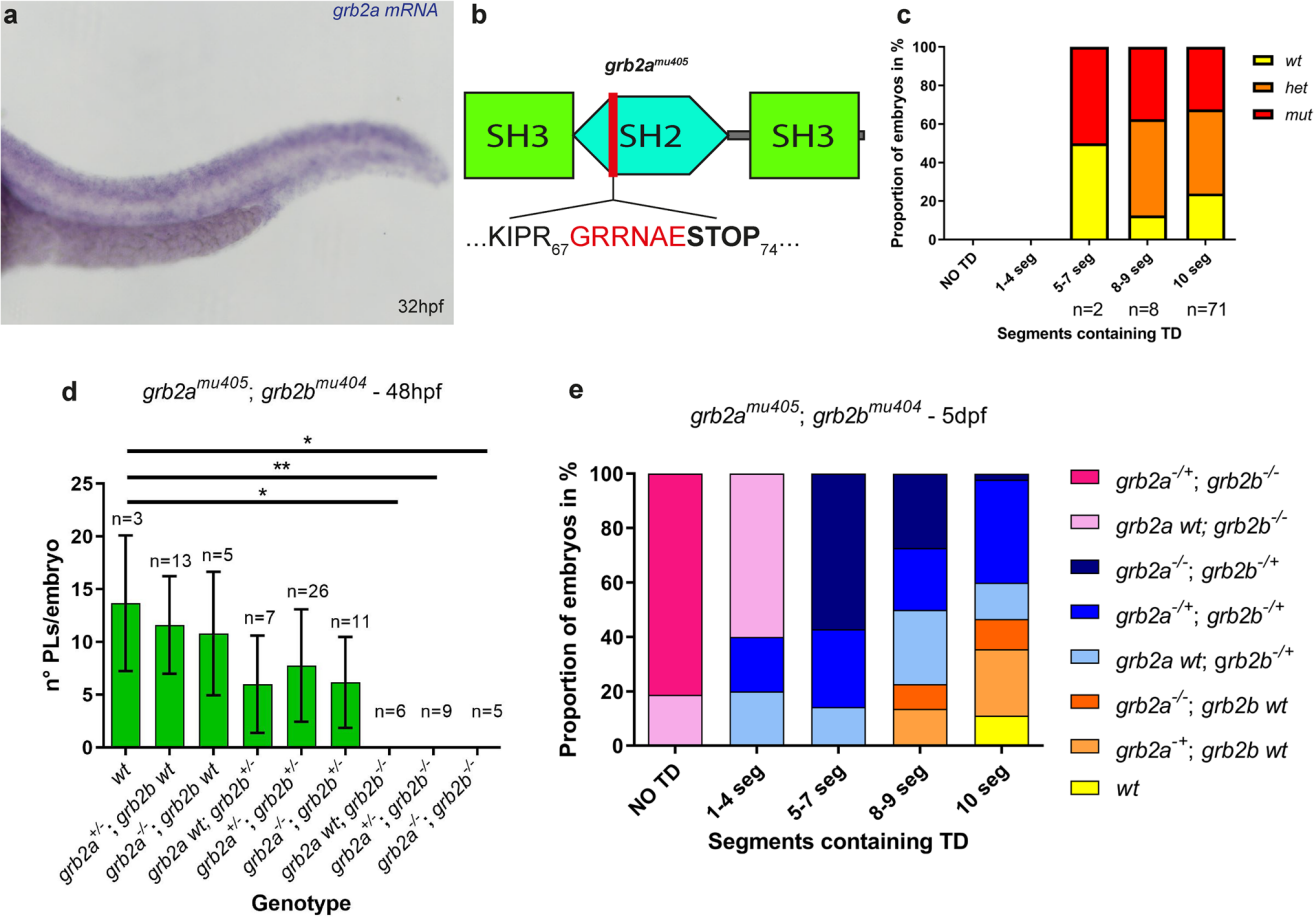
Loss of *grb2a* does not cause lymphatic defects in the trunk, but Grb2a can functionally compensate for the loss of *grb2b* upon over-expression. **a** In situ hybridization of *grb2a* shows wide expression in the zebrafish embryo at 32hpf. **b** Schematic representation of the Grb2a protein, with two SH3 domains and one SH2 domain, highlighting the − 5 bp mutation in the SH2 domain of the *grb2a*^*mu405*^ allele. **c** Quantification of TD fragments shows that *grb2a* is not essential for trunk lymphatic development. The presence of TD was quantified over 10 segments. wt: *n* = 19; *het*: *n* = 35; *mut*: *n* = 27. **d** Quantification of the number of PLs at 48hpf in the different genotypes of a *grb2a*^*mu405*^; *grb2b*^*mu404*^ double heterozygous in-cross. Lymphatic precursor cells do not form when embryos are mutant for *grb2b*, independent of the number of *grb2a* wild-type copies. * Between *wt* and *grb2a wt*; *grb2b*^*−/−*^: *P* value 0.0119 (Mann–Whitney); **Between *wt* and *grb2a*^+/−^; *grb2b*^*−/−*^: *P* value = 0.0045 (Mann–Whitney); *Between *wt* and *grb2a*^*−/−*^; *grb2b*^*−/−*^: *P* value = 0.0179 (Mann–Whitney). Note that the comparison between *wt* and each one of the other genotypes resulted in a non-significant difference. **e** Quantification of TD^+^ segments in embryos from *grb2a*^*mu405*^; *grb2b*^*mu404*^ double heterozygous parents at 5dpf. The development of the main lymphatic vessel depends on *grb2b*, and not on *grb2a*. **d**, **e** *wt*: *n* = 5; *grb2a*^+/−^; *grb2b wt*: *n* = 14; *grb2a*^*−/−*^; *grb2b wt*: *n* = 7; *grb2a wt*; *grb2b*^+/−^: *n* = 14; *grb2a*^+/−^; *grb2b*^+/−^: *n* = 25; *grb2a*^*−/−*^; *grb2b*^+/−^: *n* = 11; *grb2a wt*; *grb2b*^*−/−*^: *n* = 6; *grb2a*^+/−^; *grb2b*^*−/−*^: *n* = 13. *SH2* Src homology domain 2, *SH3* Src homology domain 3, *PL* parachordal lymphangioblast, *TD* thoracic duct. Data in **d** represent the mean ± sd

Due to genome duplication, two zebrafish orthologs for *GRB2* exist: *grb2a* and *grb2b*. As *grb2a* and *grb2b* genes and proteins share 79% and 94% identities, respectively, we wanted to address a possible involvement of *grb2a* in lymphatic development. First, an in situ hybridization for *grb2a* on 32hpf embryos was performed, and as for *grb2b*, *grb2a* was also found to be widely expressed in different tissues (Fig. 3a, Supplementary Fig. S4e–h). Second, a *grb2a* mutant allele was generated using the CRISPR/Cas9 system. This allele, *grb2a*^*mu405*^, contains a 5 bp deletion, leading to a frameshift and a predicted premature stop codon at the N-terminal part of the SH2 domain (Fig. 3b). Subsequent analysis of homozygous *grb2a*^*mu405*^ mutants did not reveal any lymphatic defects at 5dpf, as assessed by TD analysis (Fig. 3c). Additionally, we analyzed a *grb2a*^*mu405*^; *grb2b*^*mu404*^ double heterozygous in-cross to check for possible lymphatic defects in the different allelic combinations. At 48hpf, the number of PLs was significantly decreased only in embryos homozygous mutant for *grb2b*, independent of the number of wild-type *grb2a* copies (Fig. 3d). Accordingly, an impairment of TD formation was only observed when embryos lacked both copies of *grb2b*. We further noticed a weak lymphatic phenotype in embryos heterozygous for *grb2b*, but again this was independent of *grb2a* (Fig. 3e). In order to check whether Grb2a could in principle functionally replace Grb2b during lymphangiogenesis, wild-type *grb2a* mRNA was injected into eggs from a *grb2b*^*mu404*^ heterozygous in-cross. We found that providing excess *grb2a* mRNA ubiquitously was indeed sufficient to restore lymphatic structures in *grb2b*^*mu404*^ mutant embryos at 5dpf (Supplementary Fig. S5a-d). Given that Grb2a is able to functionally replace Grb2b in this over-expression setup, the *grb2b* mutant phenotype suggests that *grb2a* is normally not expressed, or not expressed in sufficiently high levels, in endothelial cells to affect lymphatic development. Taken together, *grb2b* appears to be the main player involved in lymphatic development, and, while Grb2a can compensate for Grb2b when over-expressed, it is not essential for lymphangiogenesis in the zebrafish trunk.

We additionally analyzed lymphatic structures in regions different from the trunk, such as the facial lymphatic system (FL) and the brain lymphatic endothelial cells (BLECs) in the meninges [32–35]. The development of these structures was analyzed in embryos from a *grb2a*^*mu405*^; *grb2b*^*mu404*^ double heterozygous in-cross. Embryos missing both copies of *grb2b* showed only a partial loss of FLs and BLECs structures. However, this phenotype was exacerbated when an additional copy of *grb2a* was missing, resulting in the absence of the respective lymphatic structures (Supplementary Fig. S6a–h). These results indicate that, although being dispensable for the development of the trunk lymphatics, Grb2a is required for the formation of lymphatic structures in the head.

### Grb2b is part of the Vegfc/Vegfr3 signaling axis

Previous in vitro data suggested that GRB2 constitutes a downstream effector of different tyrosine kinase receptors, including VEGFR3 [20, 36]. To establish a connection between Grb2b and the Vegfc/Vegfr3 signaling pathway in vivo, we first assessed a genetic interaction between *grb2b* and Vegfc signaling, making use of a previously published in vivo Vegfc activity assay. In this setup, forced expression of zebrafish Vegfc from the floorplate through a *shh* enhancer results in hyper-sprouting of venous endothelial cells, which is suppressed in mutant situations that impact Vegfc activity [6, 37]. When full-length Vegfc was expressed from the floorplate in wild-type and heterozygous *grb2b*^*mu404*^ embryos, a pronounced venous hyper-branching phenotype was evident at 48hpf (Supplementary Fig. S7a, b). In homozygous *grb2b*^*mu404*^ mutants, however, this dominant phenotype was strongly suppressed, which is in line with the notion that Grb2b represents a major downstream effector of Vegfc signaling (Supplementary Fig. S7).

**Fig. 4:**
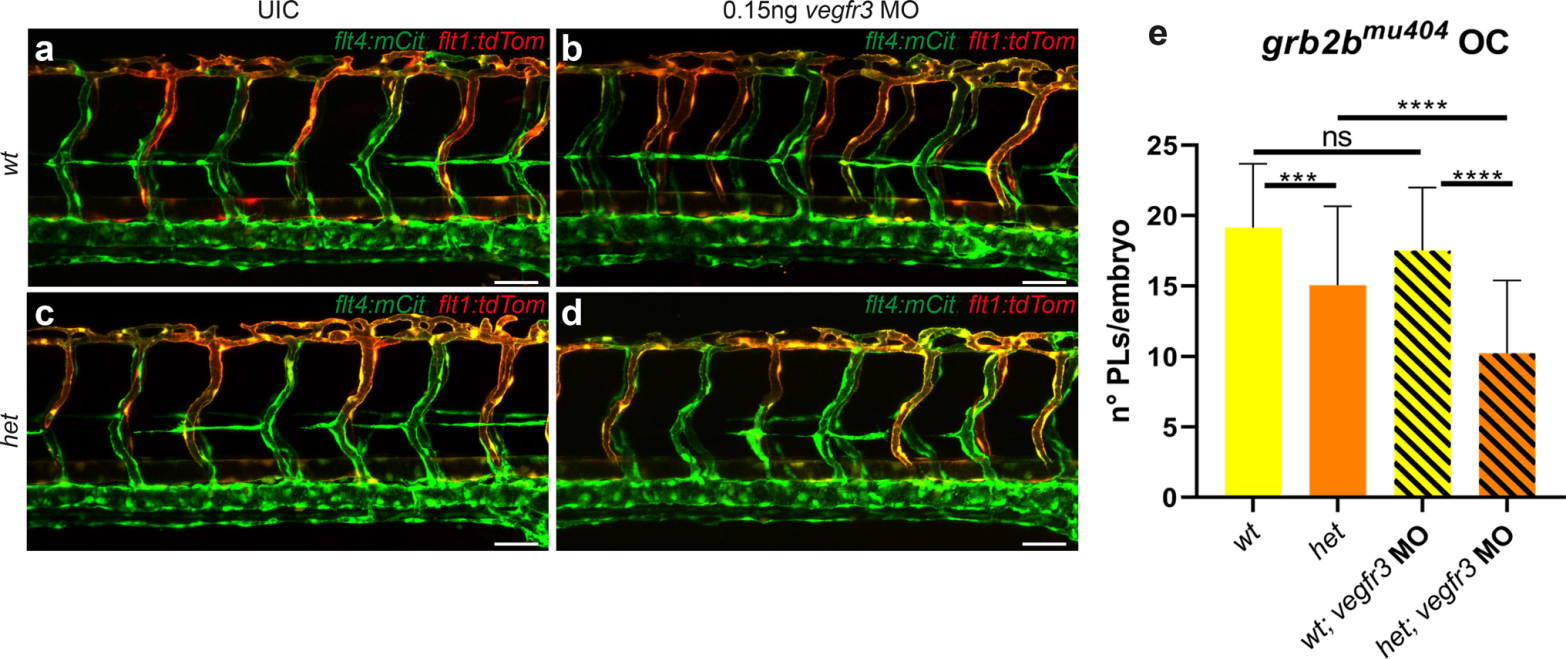
*grb2b* is a member of the Vegfc/Vegfr3 signaling pathway. **a**–**e** Injections of 0.15 ng *vegfr3* MO in embryos from a *grb2b*^*mu404*^ outcross. **a**–**d** Confocal pictures of 48hpf old embryos; *flt4:mCitrine* is shown in green and *flt1:tdTomato* in red. **e** There is no difference in the number of PLs between un-injected *wt* embryos and *wt* embryos injected with 0.15 ng of *vegfr3* MO. *grb2b*^*mu404*^ heterozygous embryos show a significant decrease in PLs after injection of 0.15 ng of *vegfr3* MO. *wt* UIC: *n* = 45; *wt* 0.15 ng *vegfr3* MO: *n* = 51; *het* UIC: *n* = 49; *het* 0.15 ng *vegfr3* MO: *n* = 43. ***Between *wt* and *het*: *P* value = 0.0002 (*t* test, two tailed); ***Between *wt* 0.15 ng *vegfr3* MO and *het* 0.15 ng *vegfr3* MO: *P* value < 0.0001 (Mann–Whitney); ****Between *het* and *het* 0.15 ng *vegfr3* MO: *P* value < 0.0001 (*t*-test, two tailed), *ns* not significant, *UIC* un-injected control, *MO* morpholino, *OC* outcross, *PL* parachordal lymphangioblast. Scale bars: 50 μm. Data in **e** are mean ± sd

Furthermore, we tested for a genetic interaction with Vegfr3 directly by injecting a sub-critical dose of a *vegfr3*-targeting morpholino (MO) into a *grb2b*^*mu404*^ outcross. Embryos were subsequently analyzed for formation of PLs at the horizontal myoseptum at 48hpf. At the injected concentration of the *vegfr3* MO, the number of lymphatic precursor cells was not altered when compared to un-injected wild-type embryos, indicating that the knockdown was not efficient enough to cause lymphatic defects on its own (Fig. 4a, b, e). However, *grb2b*^*mu404*^ heterozygous embryos injected with the same dose of *vegfr3* MO showed a significant decrease in the number of PLs compared to un-injected *grb2b*^*mu404*^ heterozygous embryos (Fig. 4c–e). This result therefore establishes a genetic interaction between *grb2b* and *vegfr3*, revealing for the first time that Grb2b acts within the Vegfr3 pathway in vivo.

### *grb2b* mutants show a decrease in endothelial cell numbers expressing Prox1 and pERK in the PCV

**Fig. 5:**
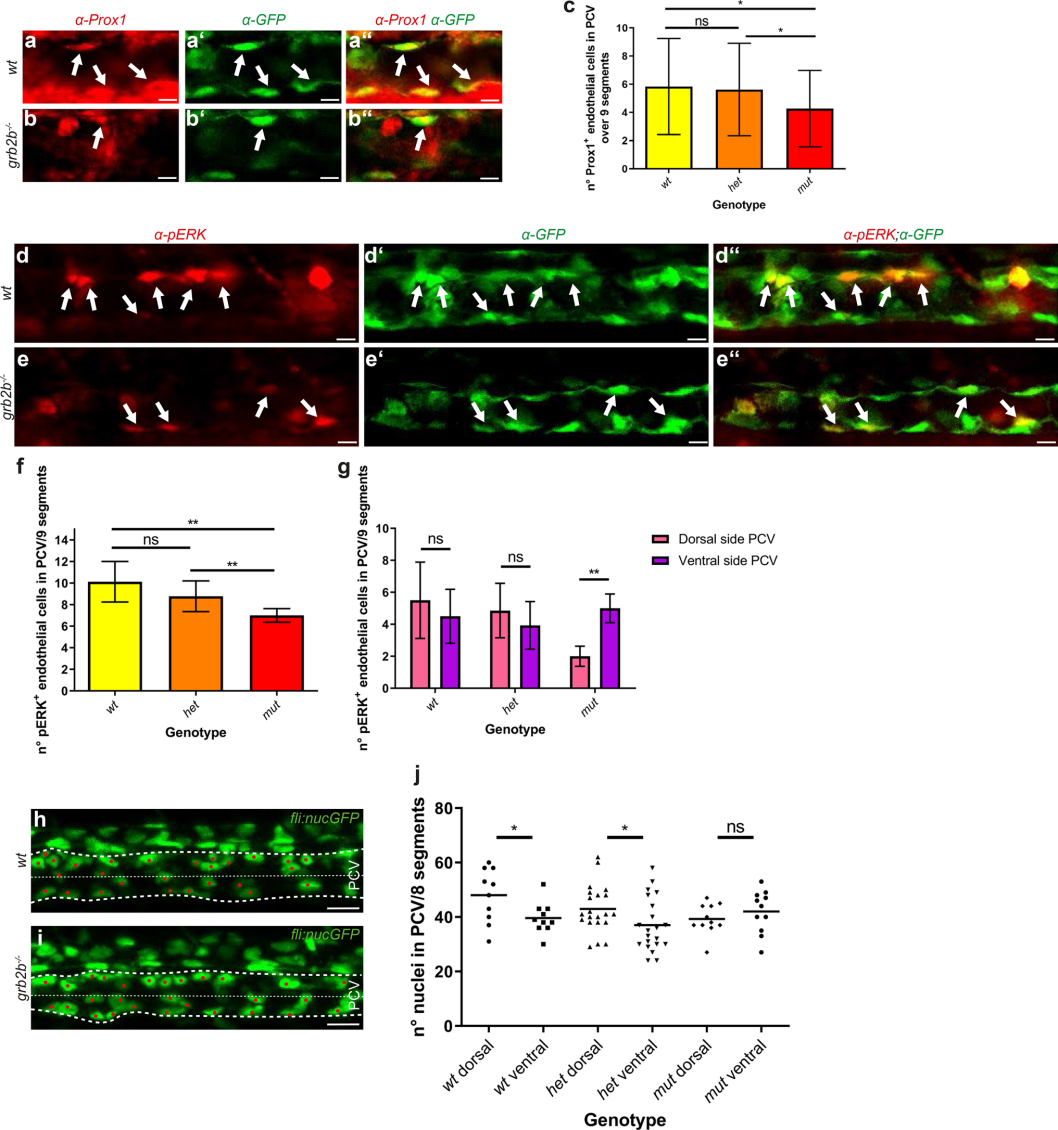
*grb2b* mutants show a defect in PCV polarization at 32hpf and exhibit decreased numbers of Prox1 and pERK positive endothelial cells. **a**–**c** The number of Prox1^+^ endothelial cells is significantly reduced in *grb2b*^*mu404*^ mutants at 32hpf. **a**–**b**ʺ Confocal projections of the PCV at 32hpf in wild-type and mutant embryos. Prox1 antibody staining is shown in red and *flt4:mCitrine* in green, with arrows pointing at cells that co-express Prox1 and Flt4. **c** Quantification of Prox1^+^ endothelial cells in the PCV at 32hpf across 9 segments. *wt*: *n* = 38; *het*: *n* = 72; *mut*: *n* = 33. *Between *wt* and *mut*: *P* value = 0.0373 (*t* test, two-tailed); *Between *het* and *mut*: *P* value = 0.0412 (*t* test, two-tailed). **d**–**f** pERK^+^ endothelial cells are reduced in numbers in *grb2b*^*mu404*^ mutants at 32hpf. **d**–**e**ʺ Confocal projections showing pERK antibody staining in red and endothelial cells in green (*flt4:mCitrine*) in the PCV at 32hpf. Arrows point at cells that express both pERK and Flt4. **f** Quantification of the number of pERK^+^ cells across 9 segments, demonstrating a decreased number in *grb2b* mutants. *wt*: *n* = 8; *het*: *n* = 14; *mut*: *n* = 6. **Between *wt* and *mut*: *P* value = 0.0013 (Mann–Whitney); ** between *het* and *mut*: *P* value 0.0036 (Mann–Whitney). **g** Moreover, pERK^+^ cells in wild type and heterozygotes are equally distributed between dorsal and ventral hemispheres of the PCV, whereas in mutants they are more concentrated in the ventral side. *wt*: *n* = 8; *het*: *n* = 14; *mut*: *n* = 6. **Between dorsal side and ventral side in *mut*: *P* value = 0.0022. **h**, **i** Confocal projections of *fli:nEGFP* wild-type and mutant embryos, highlighting the PCV between the more prominent dashed lines. The thinner dashed lines divide the PCV in two equal parts: the dorsal and the ventral side. **j** Quantifications of nuclei in the PCV at 32hpf showing that in *grb2b*^*mu404*^ mutants, endothelial cells are equally distributed between the ventral and the dorsal side. In siblings, endothelial cells are enriched in the dorsal part of the PCV. *wt*: *n* = 10; *het*: *n* = 21; *mut*: *n* = 11. *Between *wt* dorsal and *wt* ventral: *P* value 0.0327 (*t* test, two-tailed); *Between *het* dorsal and *het* ventral: *P* value  = 0.044 (*t* test, two-tailed). *ns* not significant. Scale bars: **a**–**b**ʺ: 10 µm; **d**–**e**ʺ: 10 µm; **h**, **i** 20 µm. Data in **c**, **f**, **g** are mean ± s.d. Data in **j** represent the mean with individual data points. All quantifications have been done blindly

Since Grb2b appears to act downstream of Vegfc/Vegfr3, we checked *grb2b* mutants for early Vegfc-related phenotypes that could contribute to the loss of PLs at 48hpf. It was previously reported that bi-potential precursor cells in the PCV divide asymmetrically at around 32hpf, giving rise to a cell with high Prox1a expression levels (Prox1a^high^), which has a high likelihood of developing into a PL cell, and a daughter cell with low amounts of Prox1a (Prox1a^low^), which remains in the PCV. Importantly, the induction of Prox1a expression in these venous endothelial cells seems to depend on Vegfc signaling levels [13]. To examine if Prox1a expression was altered in *grb2b* mutants, Prox1 antibody staining was performed and showed a slight, but significant decrease in the number of Prox1-positive endothelial cells in the PCV of *grb2b*^*mu404*^ mutants (Fig. 5a–c), consistent with a reduced Vegfc signaling output in the absence of Grb2b. Along the same lines, we also addressed the levels of activated ERK in *grb2b* mutants, as different in vitro studies have demonstrated that VEGFC/VEGFR3 signaling can activate ERK1/2 [16, 38] via GRB2 [20]. Antibody staining against pERK revealed significantly reduced numbers of pERK^+^ endothelial cells in the PCV of mutants compared to both wild-type and heterozygous siblings at 32hpf (Fig. 5d-f). Taken together, these results are in line with the notion that Grb2b acts downstream of Vegfc/Vegfr3 and up-stream of ERK1/2, and that the signaling output of the pathway is reduced in the absence of this critical adaptor protein.

Prior to the induction of Prox1a, the PCV undergoes a polarization event leading to a higher number of nuclei on the dorsal half of the PCV [13]. We therefore assessed whether this PCV polarization is also affected in *grb2b* mutant embryos even prior to the onset of secondary sprouting. Quantification of the number of nuclei in the PCV at 32hpf using the transgenic line *fli1a:nEGFP* demonstrated that polarization of the PCV was defective in *grb2b*^*mu404*^ mutants, as, unlike in the wild-type and heterozygous situation, more nuclei were present on the ventral than on the dorsal side of the vessel (Fig. 5h–j). Consequently, we also found an increase of pERK^+^ endothelial cells in the ventral half of the PCV when compared to the sibling situation (Fig. 5g). The total number of nuclei did not differ among the three genotypes, suggesting that the defect is not caused by aberrant endothelial proliferation (data not shown). Taken together, these results indicate an impairment in the polarization of the PCV prior to sprouting, which likely contributes to the strong lymphatic defects seen in *grb2b* mutants at 48hpf.

### Secondary sprouting is defective in *grb2b* mutants

**Fig. 6:**
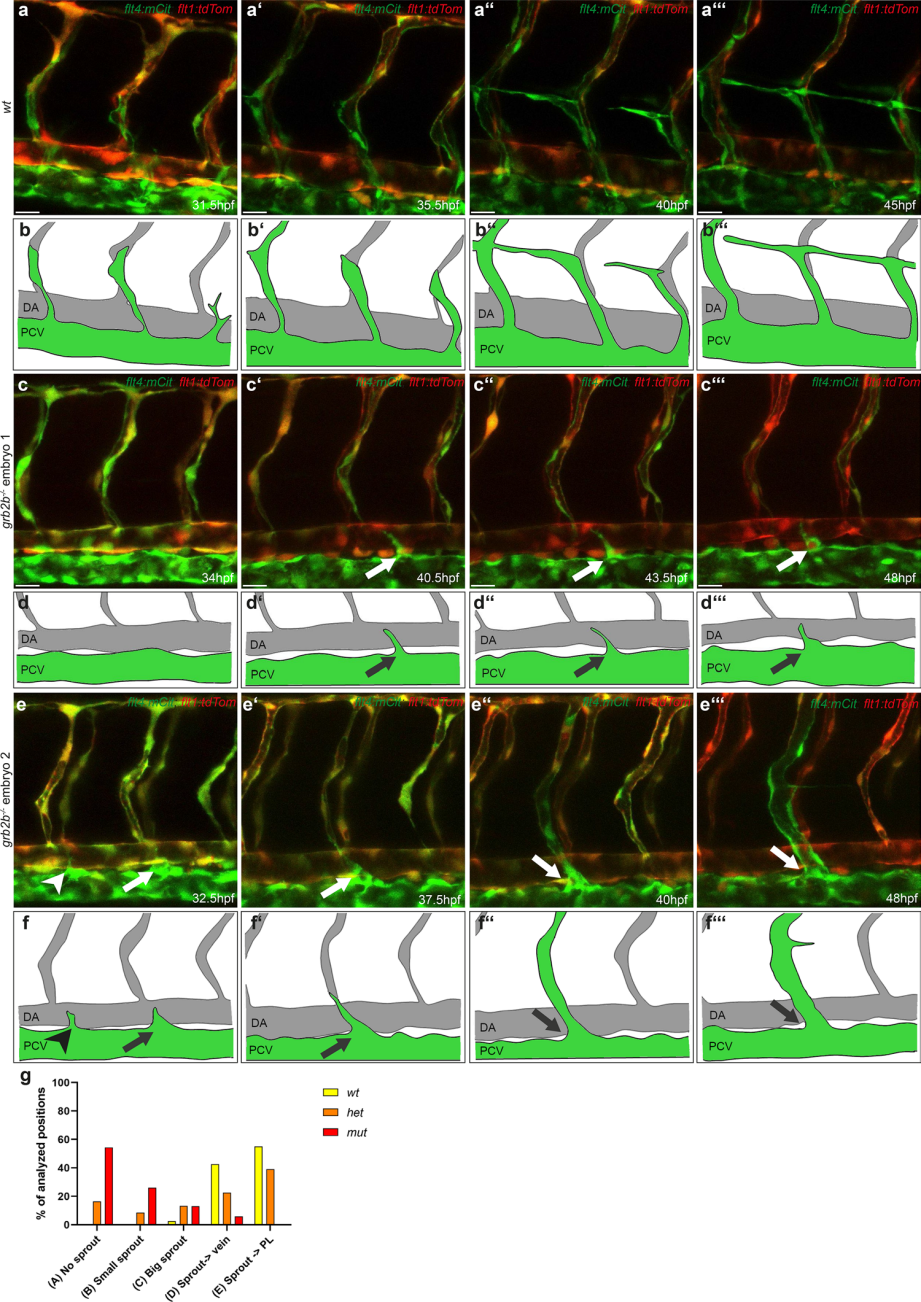
Secondary sprouting is defective in *grb2b* mutant embryos. **a**–**f**ʹʺ Still images taken from overnight videos (see Supplementary movies 1–3) and corresponding schematic cartoons of wild type (**a**–**b**ʹʺ) and *grb2b*^*mu404*^ mutant (**c**–**f**ʹʺ) embryos between 30hpf and 48hpf, showing different sprout behaviors. **a** In wild-type embryos, cells sprout from the PCV and either form PLs at the HM or remodel an intersegmental artery into a vein. **c**, **e** Still images of two different *grb2b* mutant embryos. The arrow in **c**ʹ–**d**ʹʺ highlights a cell attempting to sprout from the PCV and extending towards an aISV, but failing to establish a stable connection. The arrowhead in **e** and **f** points at an endothelial cell with a small filopodium that retracts. The arrows in pictures from **e** to **f**ʹʺ point at an endothelial sprout that reaches an aISV, establishes a stable connection and remodels the artery into a vein. In all images *flt4:mCitrine* is shown in green and *flt1:tdTomato* in red. **g** Quantification of the different sprout behaviors observed between 31 and 48hpf (indicated as percentage). For quantification, both sides of a four segments stretch per embryo were analyzed. *wt*: *n* = 5, *het*: *n* = 16, *mut*: *n* = 23. Scale bars: 25 µm. Data in *d* are mean ± sd

In order to better understand the *grb2b* phenotype, we performed time-lapse imaging between 30 and 48hpf to examine the initial phase of lympho-venous sprouting and migration in more detail and to address the question whether additional defects during this phase might impact the formation of PLs and vISVs (Fig. 6a–fʹʺ). We followed the sprouting activity at a total of 8 positions per embryo (four consecutive segments, bilateral) and grouped the observed cell behaviors into five categories: (A) no sprouting activity, (B) minor sprouting activity (only extension of cell protrusions but no egression from the PCV), (C) major sprout activity (cell emerging from the PCV, but not forming either a vISV or a PL), (D) sprout remodeling an artery into a vein, and (E) sprout giving rise to a PL (Fig. 6g). In wild-type and *grb2b* heterozygous cases, most positions gave rise to secondary sprouts that either formed vISVs (42.5% in wt and 22.7% in het; category D) or PL cells (55% in wt and 39.1% in het; category E). In *grb2b* mutant embryos, however, 54.4% of the analyzed positions did not show detectable sprouting activity (category A), while in 39.1% of the cases only a reduced sprouting activity (categories B + C) was evident. In addition, we found at 6% of the imaged positions a sprout that successfully remodeled an ISV (category D) while only one out of 184 quantified positions gave rise to a sprout that migrated to the HM to form a PL (0.5%, category E). Therefore, the overall number of sprouts formed in *grb2b* mutants was found to be reduced. Thus, a lack of Grb2b activity strongly impairs the sprouting and migration capacity of venous endothelial cells.

**Fig. 7:**
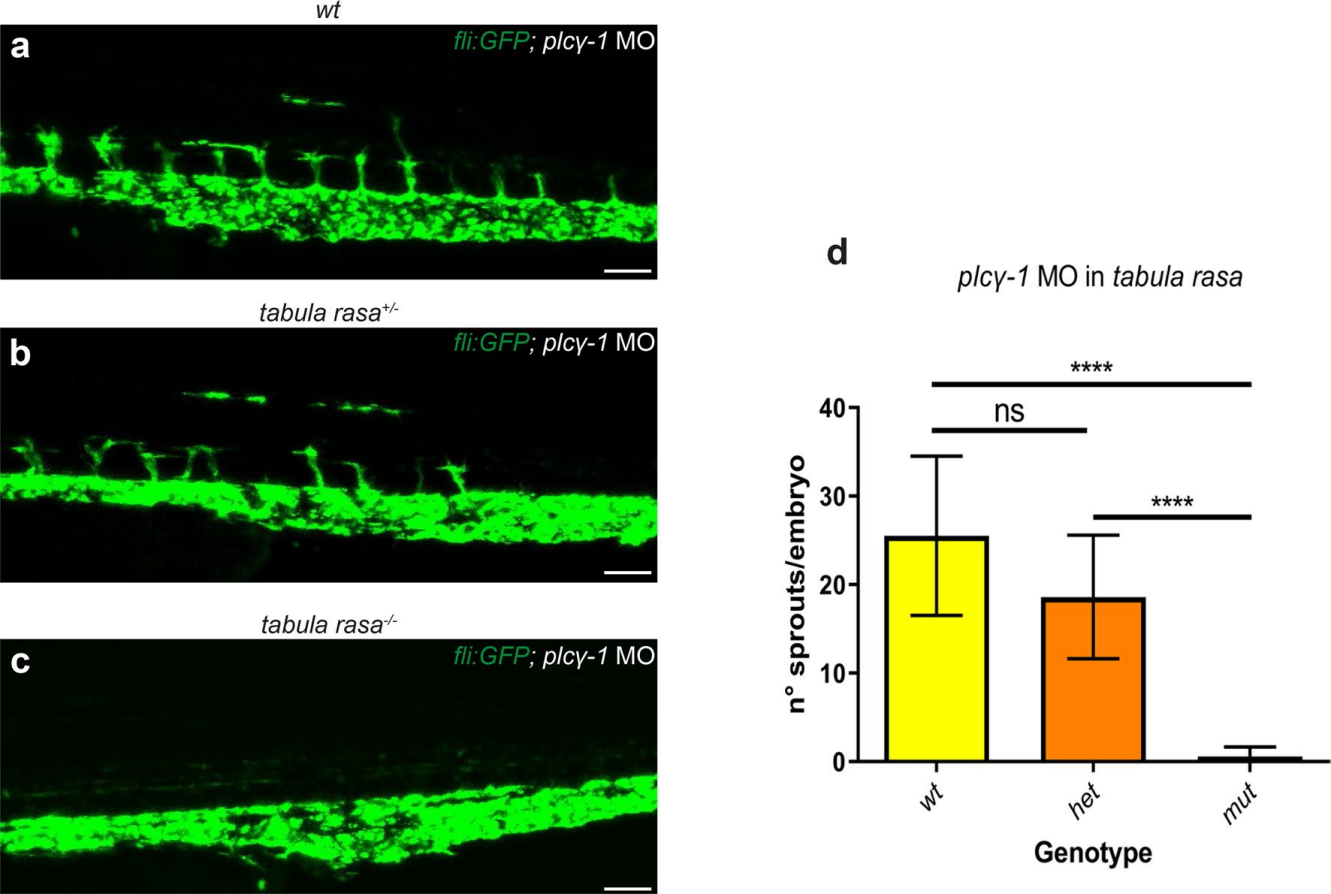
In the absence of arteries, cells fail to sprout in *grb2b* mutants. **a**–**c** Confocal projections of *plcγ-1* MO injected embryos. All vessels are shown in green (*fli:GFP*). **d** Sprout quantification at 48hpf of embryos from a *tabula rasa* in-cross, injected with a *plcγ-1* MO. *plcγ-1* MO injection inhibits primary sprouting, allowing quantification/visualization of secondary sprouts in absence of arteries. The number of lympho-venous sprouts is significantly decreased in *tabula rasa* mutants. *wt*: *n* = 4; *het*: *n* = 32; *mut*: *n* = 24. ****Between *wt* and *mut*: *P* value  < 0.0001 (Mann–Whitney); ****Between *het* and *mut*: *P* value < 0.0001 (Mann–Whitney); *ns* not significant. *MO* morpholino. Scale bars: 50 µm. Data in **d** are mean ± sd

As intersegmental arteries play a role in vein formation in the wild-type situation [14], we wanted to investigate the sprouting behavior in *grb2b* mutants in the absence of arteries. Injection of a *phospholipase C gamma-1* (*plcγ-1*) morpholino (MO) [39] resulted in the absence of intersegmental vessels in both *tabula rasa* mutants and siblings (Fig. 7a–d). In this set-up, the number of sprouts emerging from the PCV was quantified at 48hpf. The analysis revealed a drastic decrease of sprouts in homozygous *grb2b* mutants compared to siblings, suggesting that in the absence of intersegmental arteries almost no stable sprouts could be established.

**Fig. 8:**
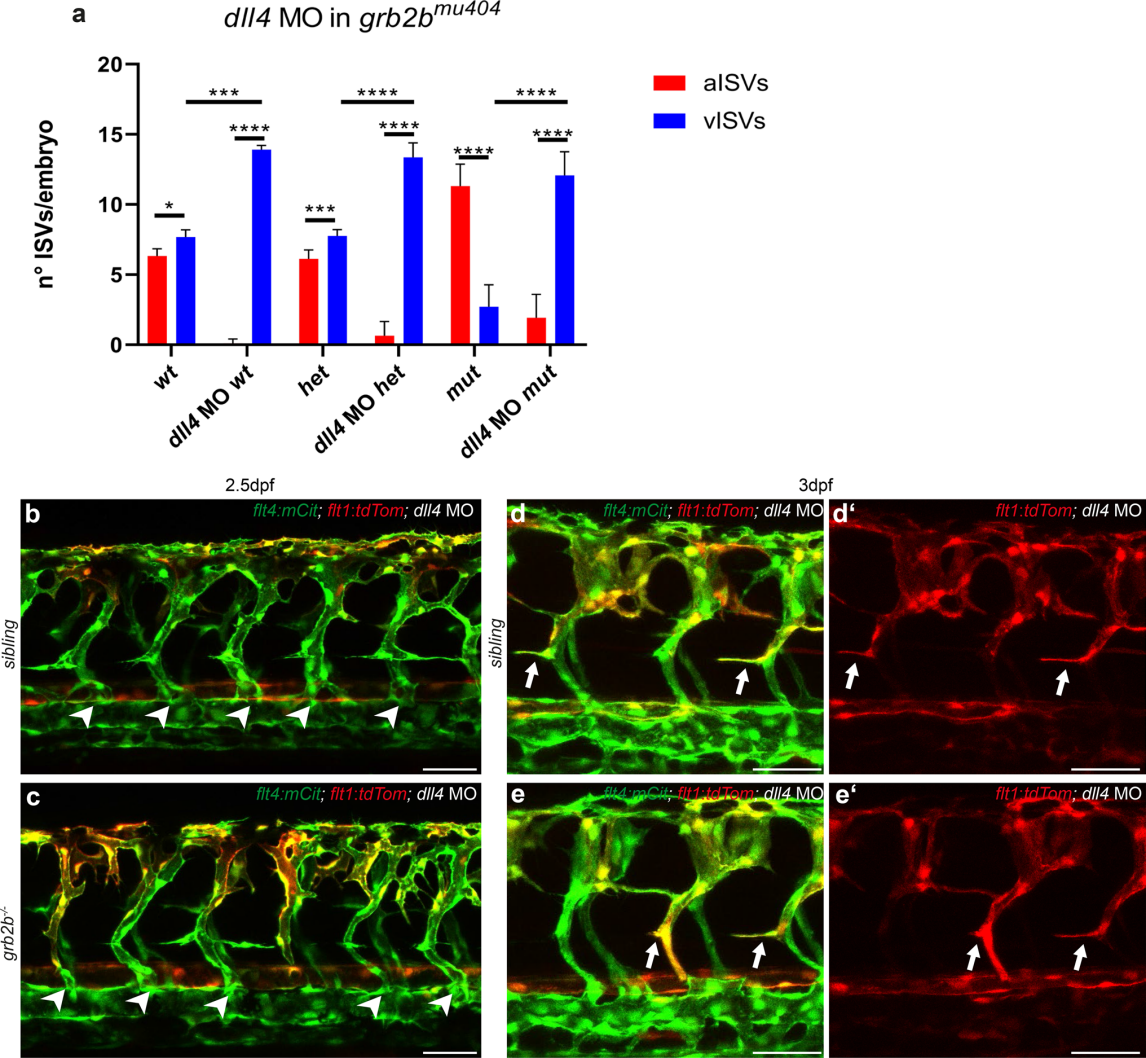
*grb2b* mutants show an increase in vISVs upon *dll4* knockdown. **a** Quantifications of aISVs and vISVS at 2.5dpf in un-injected control and *dll4* MO injected embryos from *grb2b*^*mu404*^ heterozygous parents. 14 ISVs per embryo were analyzed. *Between aISVs and vISV in *wt*: *P* value 0.013 (Mann–Whitney); ****Between aISVs and vISV in *dll4* MO *wt*: *P* value < 0.0001 (Mann–Whitney); ***Between aISVs and vISV in *het*: *P* value 0.0009 (Mann–Whitney); ****Between aISVs and vISV in *dll4* MO *het*: *P* value < 0.0001 (Mann–Whitney); ****Between aISVs and vISV in *mut*: *P* value < 0.0001 (Mann–Whitney); ****Between aISVs and vISV in *dll4* MO *mut*: *P* value < 0.0001 (Mann–Whitney); ***Between vISVs *wt* and vISVs *dll4* MO *wt*: *P* value = 0.0001 (Mann–Whitney); ****Between vISVs *het* and vISV in *dll4* MO *het*: *P* value < 0.0001 (Mann–Whitney); ****Between vISVs *mut* and vISV in *dll4* MO *mut*: *P* value < 0.0001 (Mann–Whitney). **b**–**e**ʹ Confocal pictures of *grb2b* mutant or sibling embryos at 2.5dpf (**b**, **c**) and 3dpf (**d**–**e**ʹ), injected with *dll4* MO. Veins are shown in green (*flt4:mCitrine*) and arteries in red (*flt1:tdTomato*). Arrowheads highlight vISVs while arrows mark protrusions from ISVs extending towards the HM. *aISV* arterial intersegmental vessel, *vISV* venous intersegmental vessel, *MO* morpholino. Scale bars: 50 µm. Data in a represent mean ± sd

Since *grb2b* mutants predominantly gave rise to smaller and more transient sprouts, we investigated in more detail their capacity of reaching an ISV to form an intersegmental vein. It was recently shown that the specification of intersegmental arteries and veins is already predetermined in a Notch-mediated fashion within intersegmental vessels before the onset of secondary sprouting [14]. ISVs with high Notch activity are pre-specified to give rise to an artery, while ISVs with low Notch signaling are specified to become intersegmental veins. In consequence, knockdown of the Notch ligand Dll4 results in an increase of veins at the expense of arteries [40], since under these conditions Notch activation in all ISVs is reduced. In order to assess a situation in which almost all intersegmental vessels would be predetermined to give rise to intersegmental veins, we injected a *dll4* MO into an in-cross of *grb2b*^*mu404*^ heterozygotes. Embryos were imaged at 2.5dpf to allow quantification of aISVs and vISVs. As expected, injection of *dll4* MO into wild-type embryos resulted in a dramatic increase in the number of vISVs with very few remaining aISV. In *grb2b* mutant embryos, significantly, knock-down of *dll4* also led to a strong increase in the number of vISVs as 86.3% of all ISVs were successfully remodeled into a vein (on average, only 2 aISVs out of 14 quantified ISVs remained) (Fig. 8a-c). In addition to this robust rescue of vein formation as such, we noticed the appearance of cellular protrusions stemming from ISVs and extending towards the HM in all *dll4* morphant situations. These aberrant protrusions were of variable length and seemed to be connected to the respective ISV even at 3dpf (Fig. 8d–e'). Furthermore, they were often positive for the arterial marker *flt1:tdTomato* (Fig. 8d, e). We therefore concluded that these structures are likely a secondary effect of hyper-sprouting ISVs upon *dll4* knock-down and hence we did not consider them as a rescue in PL formation, a notion which is supported by previous work that reported a strong reduction in the number of PL cells in *dll4* morphants [41]. Taken together, these results suggest that, in principle, *grb2b* mutant venous sprouts are capable of forming veins, but that this step is suppressed by wild-type Dll4 signalling levels.

## Discussion

The essential role of the Vegfr3 pathway for lymphangiogenesis is conserved among vertebrates. While work in the past few years has provided a good understanding of the extracellular proteins that aid in producing processed and biologically active Vegfc protein, and in the functionality of different Vegfr3 domains, our insights into the intracellular events upon Vegfr3 activation are comparatively limited. Here, we characterize a mutant in the zebrafish *grb2b* gene. In contrast to other mutants affecting the Vegfc/Vegfr3 signaling axis (which usually affect venous and lymphatic sprouting equally strong), *grb2b* mutants lack lymphatic structures while forming some veins, allowing to distinguish between the effect of low Vegfr3 signaling activity on venous versus lymphatic cells in zebrafish.

The mutant discovered in the forward genetic screen harbors a point mutation in the *grb2b* gene, leading to a predicted amino acid change at position 48. From the crystal structure analysis of the human GRB2 protein, several residues in the first SH3 domain, including Phe47 and Pro49, were found to be important for the protein–protein interaction site [30]. Considering that all three amino acids in position 47, 48 and 49 are conserved in human, mouse and zebrafish, the mutation in the *tabula rasa* allele is therefore likely to interfere with the binding of downstream ligands (such as Sos). An additional *grb2b* loss-of function allele, *grb2b*^*mu404*^, does not show any significant phenotypic difference to the *tabula rasa* allele, thus indicating that the *tabula rasa* allele also represents a loss-of-function situation. GRB2 is known to be expressed in many tissues in human and mice [17], a finding that is reciprocated in zebrafish. Murine embryos that lack Grb2 die very early, at E4.5, because of a failure in endoderm differentiation [42]. Surprisingly, given the many tyrosine kinase receptors with which Grb2 is assumed to interact, *grb2b* zebrafish mutants are viable up to 5dpf and they do not show any other overt phenotypical defect except for vascular development. A possible explanation could be maternal contribution, and indeed, *grb2b* is both zygotically and maternally expressed [43]. Maternally provided mRNAs could compensate for zygotic *grb2b* loss-of-function in different tissues, allowing fish embryos to survive the first few days. Moreover, the likewise widely expressed Grb2a could contribute to survival by compensating in tissues other than endothelial cells in the trunk. Indeed, *grb2a*/*grb2b* homozygous mutants are not viable at 4dpf, indicating a role of Grb2a in the early larva outside the vascular system. In addition, Grb2a is able to partially compensate for Grb2b loss-of-function in FLs and BLECs, as these structures are developing later then the lymphatics in the trunk and therefore are probably less dependent on maternal contribution. Finally, the fact that *grb2b* mutants show a stronger phenotype in the lymphatics in the trunk compared to the head is in accordance with previous data, showing that an impairment of a specific region of the tyrosine kinase domain of Vegfr3, leading to a decrease in ERK signaling, is affecting TD, but not FLs formation [16]. The exceptional situation that a critical Vegfr3 pathway component is required in ECs, but appears to be dispensable in (most) other tissues, allowed the study of the role of this particular adaptor protein in venous sprouting and lymphangiogenesis.

From in vitro data, GRB2 has been suggested to be part of the VEGFC/VEGFR3 signaling pathway [20]. GRB2 was shown to directly bind VEGFR3 protein via protein blotting analysis from HUVEC (human umbilical vein endothelial cell) extracts [20], but an in vivo demonstration of Grb2 acting in concert with VEGFR3 had been missing. By injecting carefully titrated amounts of *vegfr3* MOs, we provide such demonstration: simultaneous lowering of Vegfr3 activity leads to an exacerbation of the lymphatic phenotype in *grb2b* heterozygous embryos, while no effect is noticed in wild-type embryos.

Members of the Vegfc/Vegfr3 pathway have been previously analyzed in zebrafish. Mutants in *vegfc, ccbe1* and *adamts3; adamts14* all lack lymphatics structures (PLs and TD), and vISVs fail to develop at 48hpf [4, 6, 10]. Significantly, and in contrast to the above genes, embryos deficient of Grb2b are also missing lymphatic vessels, but they retain a considerable number of veins. This unique characteristic is likely explained by the presence of additional pathways downstream of Vegfc/Vegfr3, the most likely one being PI3K/Akt [38, 44], which is Grb2b independent and presumably still active in endothelial cells that retain a certain protrusive and proliferative activity in *grb2b* mutants. Based on in vitro data, the MEK/ERK pathway is known to be activated downstream of VEGFR3 homodimers, leading to cell proliferation and migration [44]. In mice, ERK signaling was shown to be linked to lymphatic development [45]. It regulates SOX18 and PROX1 expression in endothelial cells in the vein leading to lymphatic commitment [45]. Moreover, abnormalities in the Ras/mitogen-activated protein kinase (MAPK) pathway result in developmental disorders in human, including lymphedema [45, 46]. In zebrafish, ERK was shown to be the essential downstream effector of Vegfr3 signaling for differentiation and sprouting of future lymphatic endothelial cells in the trunk [16]. Here, we show that *grb2b* mutants have a decreased number of pERK^+^ endothelial cells at 32hpf and that most of them are located in the ventral side of the PCV. Grb2b is therefore needed as an upstream moderator of these downstream signaling events for a balanced activation of the MEK/ERK cascade, which leads to a correct migration of endothelial cells within the vein prior to sprouting.

The main reason why *grb2b* mutants lack lymphatics appears to be a defect in secondary sprouting. In the absence of Grb2b, sprouts fail to emerge from the PCV in 54.5% of cases, and in 39% of cases only thin filopodia or bigger sprouts form, but without generating a functional outcome such as vein or PL formation. Previous work suggested that after the PCV undergoes a ventral-to-dorsal polarization, Prox1a expressing cells divide asymmetrically within the PCV, giving rise to one cell with high Prox1a expression levels that is subsequently sprouting from the vein and gives rise to a PL cell in the vast majority of cases [13]. *grb2b* mutant embryos show a 27% decrease in Prox1^+^ cells in the PCV and polarization is defective at 32hpf, as endothelial cells are equally distributed within the PCV, while the total number of nuclei is unchanged. These data suggest an impairment in cell re-arrangement rather than proliferation. Moreover, the decrease in Prox1 expression in *grb2b* mutants correlates with a similar phenotype in Vegfc and Vegfr3 morphants [13], supporting the notion that Grb2b acts within this pathway and is likewise involved in Prox1 induction. However, the 27% decrease in Prox1 expression is unlikely to account for the almost complete lack of PLs and for the increase in aISV/vISV ratio, and therefore other events must be influenced by Grb2b.

**Fig. 9:**
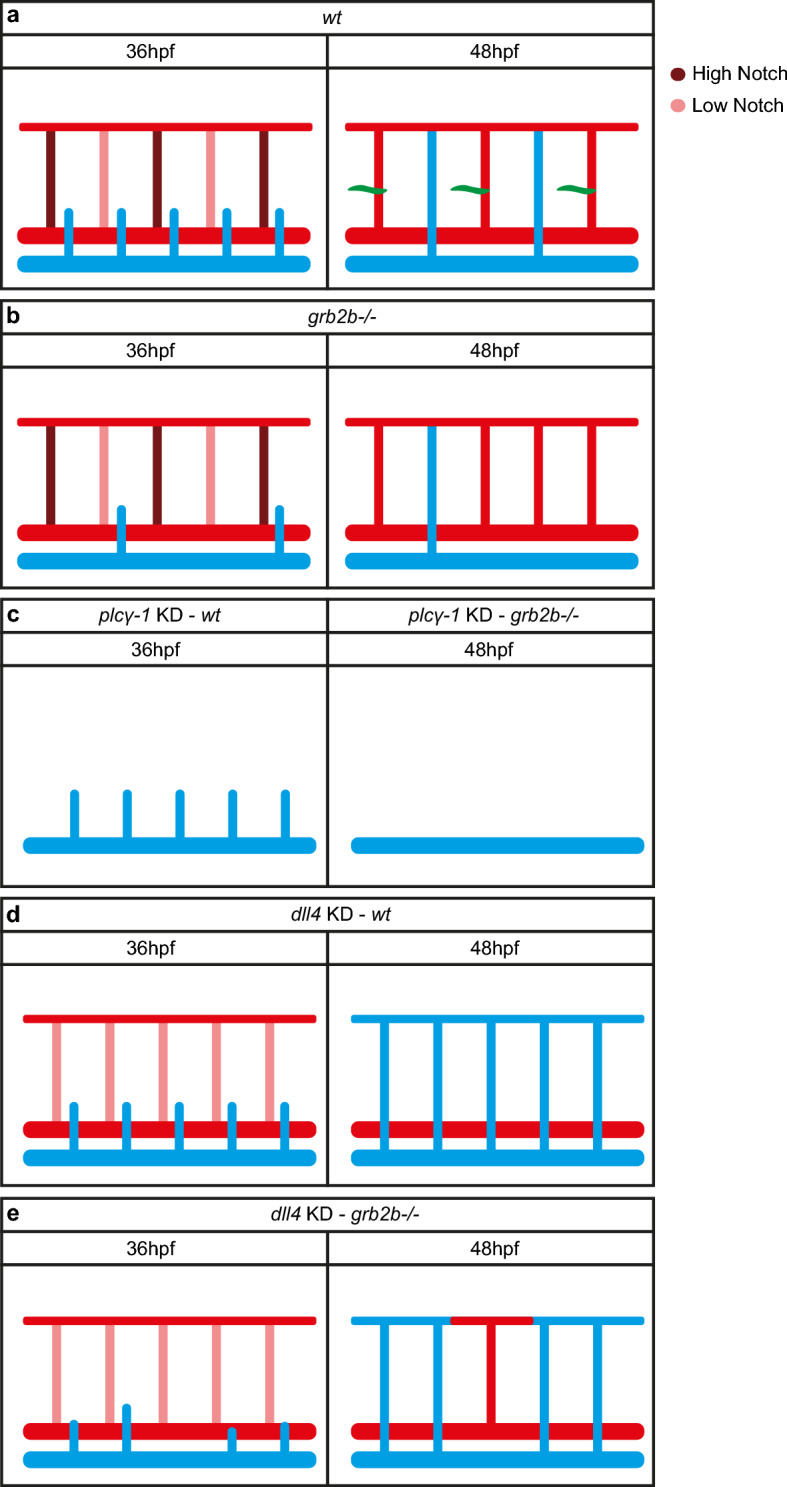
*grb2b* is essential for cells to sprout from the PCV and for PL formation, but not for vein formation. **a** In wild-type embryos, secondary sprouts are either forming PLs, shown in green (if they are close to an aISV with high Notch signaling levels), or a vISV (in case the aISV has low levels of Notch activity). **b** *grb2b* mutant embryos show defective secondary sprouting. If a cell protruding from the PCV encounters an intersegmental artery with high Notch levels, then it will retract towards the PCV, not being able to migrate to the HM. If the cell makes contact with a low Notch signaling artery, it will form an intersegmental vein. **c** Upon *plcγ-1* knockdown, no intersegmental arteries develop. In wild-type embryos sprouts normally migrate out from the PCV, whereas no stable sprout is detected at 48hpf in *grb2b* mutants. **d**, **e** Upon *dll4* knockdown most of aISVs are remodeled into vISVs in both wild-type and *grb2b* mutant embryos, indicating that a connection between a cell in the PCV and the intersegmental artery is sufficient for a venous endothelial cell to form a vein

Recently, it was postulated that an ISV-intrinsic pre-pattern, rather than a specification event within secondary sprouts themselves, has a critical influence on sprout behavior and ultimately cell fate decision [14]. Endothelial cells from the PCV migrate out to form a connection with an aISV and then either detach again in order to migrate to the HM, or they build a stable, lumenized connection and remodel the artery into a vein. This process depends on Notch signaling levels within the aISVs [14], with low arterial Dll4 levels allowing vein formation, while high levels ultimately favor PL formation. We extend this model and demonstrate that different levels of Grb2b activity (and therefore presumably different levels of Vegfc signaling) are required for venous and lymphatic sprout behavior. In the absence of Grb2b, embryos are unable to form PLs, but a fraction of venous sprouts makes physical connections to nearby arteries, leading to the formation of several veins (6%) (Fig. 9a, b). Our interpretation is that sprouts giving rise to vISVs do not have to move away from the PCV completely, and those secondary sprouts that manage to establish critical connectivity to Dll4-low aISVs will have, in some cases, the ability to establish a stable connection and to generate a vein. In *grb2b* mutants, alternative Vegfr3 downstream pathways (e.g., PI3K signaling) are apparently sufficient to provide enough signaling input to allow this step to happen. In the absence of any ISVs that secondary sprouts could connect to (*plcγ-1* morphants), *grb2b* mutants fail to form persistent sprouts, suggesting that the connection to an ISV is required to stabilize the venous sprouts in the absence of Grb2b (Fig. 9c).

Lymphatic sprouts, however, which connect to a Dll4-high aISV, would have to migrate out of the PCV in order to reach the HM, and for this step Grb2b activity is more critically required. Indeed, a role for GRB2 in cell migration has been suggested based on in vitro data [47]. After inducing angiogenesis in HUVECs, cells showed an increased migratory behavior, which could be suppressed by a GRB2 antagonist [47]. The observation of a complete failure in PL formation, even when arteries are present and while some veins are being formed, points at a higher requirement for Grb2b in future PL cells versus venous sprouts.

In the absence of Notch signaling, in otherwise wild-type embryos, most aISVs are converted into veins most likely due to the lack of a repulsive force or the presence of an attractive force within ISVs (Fig. 9d) [41]. If both Grb2b and Notch signaling are missing, almost all cells that manage to sprout from the PCV are able to remodel aISVs: upon *dll4* knockdown, *grb2b* mutants developed veins in 85% of the observed cases (Fig. 9d). Here we see a partial rescue of the number of sprouts, which is in line with the notion that the reduced Vegfr3 signaling levels in *grb2b* mutants might only be permissive for the generation of sprouts that are able to connect to a nearby ISV but not for sprouts that have to migrate all the way up to the horizontal myoseptum to give rise to a PL cell.

In conclusion, we have shown a severe impairment of the lymphatic vasculature due to a loss-of-function mutation in the *grb2b* gene. Grb2b is not only needed for the formation of lymphatic precursor cells, but also for the correct sprouting of endothelial cells in the PCV. Alternative Vegfr3 downstream signaling pathways enable a fraction of venous sprouts to form veins in *grb2b* mutants, but apparently higher levels of Vegfr3 signaling outputs are required in PLs. Whether higher Grb2b activity in future PLs is an intrinsic property of lymphatic sprouts, or whether it is a feature depending on interaction with pre-patterned aISVs remains to be determined.

## Supplementary Information

Below is the link to the electronic supplementary material.

Supplementary Figure 1. *tabula rasa* links to a genomic interval on Chromosome 3. (a) Linkage analysis of each chromosome for the *tabula rasa* mutation based on next-generation sequencing data from pooled mutant and sibling DNA. Homozygosity mapping taking either all identified SNPs into account (green lines) or employing only SNPs that appeared to result from the ENU treatment of the screen fish (blue lines) indicated a possible linkage of the *tabula rasa* mutation to chromosome 3. (b) By conventional genetic linkage analysis using polymorphic markers, linkage to chromosome 3 was verified and the mutation was mapped to a region between an informative SNP in the *wnk4b* gene and the marker z10805 (position 22.75Mb and 27.32Mb according to the genome assembly Zv9). Candidate mutations in the region were subsequently identified employing the next-generation sequencing data. Electronic supplementary material 1 (PDF 387 kb)

Supplementary Figure 2. The *tabula rasa* lymphatic phenotype is rescued by *grb2b* mRNA injections. (a) TD quantification in 10 trunk segments of embryos from a *tabula rasa* in-cross as un-injected control (UIC). Most of the mutants do not develop a TD or they only have few TD fragments. wt: n=19, het: n=43, mut: n=23. (b) Embryos from a *tabula rasa* in-cross injected with 80pg of *grb2b* mRNA showing a rescue of the TD defects in mutant embryos. wt: n=18, het: n=46, mut: n=24. TD: thoracic duct. Electronic supplementary material 2 (PDF 171 kb)

Supplementary Figure 3. The *grb2b*^*mu404*^ allele causes defects that are comparable to the *tabula rasa* allele. (a) PL cells are significantly decreased in *grb2b*^*mu404*^ mutants compared to wild types and heterozygotes at 48hpf. wt: n= 12; het: n= 51; mut: n= 25. ** Between wt and het: P value 0.0033 (Mann–Whitney). ****Between wt and mut: P value <0.0001 (Mann–Whitney). **** Between het an mut: P value <0.0001 (Mann–Whitney). (b) In *grb2b*^*mu404*^ mutants, the number of vISVs is significantly reduced compared to aISVs. The total number of quantified ISVs per embryo was 40. wt: n= 6; het: n= 27; mut: n= 10. **** Between aISVs mut and vISVs mut: P value <0.0001 (t test, two-tailed). **** Between vISVs wt and vISVs mut: P value <0.0001 (t test, two-tailed). **** Between vISVs het and vISVs mut: P value <0.0001 (Mann–Whitney). (c) TD fragments are missing in *grb2b*^*mu404*^ mutant embryos at 5dpf, as shown in the quantification. Segments containing TD were quantified over the length of 10 somites. PL: parachordal lymphangioblast, TD: thoracic duct, aISV: arterial intersegmental vessel, vISV: venous intersegmental vessel; ns: not significant. Data in a, b are mean ± s.d. Electronic supplementary material 3 (PDF 160 kb)

Supplementary Figure 4. Expression pattern of *grb2b* and *grb2a* mRNA during venous sprouting. (a-g) In situ hybridization against *grb2b* on wild-type embryos at 32hpf. Within the trunk, *grb2b* mRNA can be detected in various tissues with the strongest expression being evident within the central nervous system (arrows) and the distal part of the pronephros (arrow head) (a-c). In the head region, *grb2b* is ubiquitously expressed (d). (e-h) Detection of *grb2a* mRNA in wild-type embryos at 32hpf. As for *grb2b*, *grb2a* transcripts can be detected in various different tissues within the trunk (e-g) and head region (h) with a prominent expression within the spinal cord (arrows). Scale bars in a, g, e, h: 100µm; in b, c and f, g: 50µm. Electronic supplementary material 4 (PDF 448 kb)

Supplementary Figure 5. *grb2a* can compensate for the loss of *grb2b* function in trunk lymphatics. (a,b) Confocal projections of *grb2b*^*mu404*^ mutant embryos as un-injected control (UIC, a) and injected with grb2a mRNA (b). *flt4:mCitrine* is shown in green, *flt1:tdTomato* in red. Note the absence of TD in UIC embryos (asterisk) and the presence of TD upon *grb2a* mRNA injection (arrows). (c,d) Quantification of TD formation in embryos from a *grb2b*^*mu404*^ in-cross that were not injected (c) (wt: n=20, het: n=41, mut: n=12) or that were injected with 200pg of *grb2a* mRNA (d) (wt: n=26, het: n=43, mut: n=22) indicates that Grb2a can rescue the *grb2b* mutant TD phenotype. TD: thoracic duct. Scale bars: 50µm. Electronic supplementary material 5 (PDF 333 kb)

Supplementary Figure 6. *grb2a* and *grb2b* are required for the development of the facial lymphatic system and of brain lymphatic endothelial cells. (a-f) Confocal projections of the zebrafish head at 4dpf in embryos with the indicated genotypes. Veins and lymphatics are shown in green (*flt4:mCitrine*) and arterial blood vessels in red (*flt1:tdTomato*). Arrows point at the different structures of FLs and arrowheads highlight BLECs. Asterisks mark the absence of those structures. (g,h) Quantification of FLs and BLECs structures in a *grb2a*^*mu405*^; *grb2b*^*mu404*^ double heterozygous in-cross at 4dpf. The phenotypes have been divided into three categories: normal, when all structures are present; partial, when some structures are not developed; absent, when the respective structures are missing completely. Note that within the same embryo, BLECs and FLs were mostly affected to a similar extent. Double homozygous embryos were not incorporated into the analysis since they either did not survive until 4dpf or they showed signs of tissue necrosis. FLs: facial lymphatics, BLECs: brain lymphatic endothelial cells. Scale bars: 50µm. Electronic supplementary material 6 (PDF 1036 kb)

Supplementary Figure 7. Grb2b acts downstream of the Vegfc/Vegfr3 pathway. (a-c) Confocal projections of *shh:vegfc-IRES-mTurquoise;* *grb2b*^*mu404*^ wild-type, heterozygous and homozygous embryos. The over-expression of Vegfc in the floorplate causes a dominant hyper-branching of ISVs (arrows in a, b), which is strongly suppressed by a complete loss of *grb2b* (marked by asterisk in c). (d) Quantification of the total vessel area in the dorsal aspect of ISVs in *grb2b*^*mu404*^ mutants or siblings expressing the *shh:vegfc-IRES-mTurquoise* transgene, showing a significant decrease of venous hyper-sprouting in embryos lacking both functional copies of *grb2b*. Siblings: n= 5; mut: n= 9. *** Between siblings and mut: P value =0.001 (Mann–Whitney). Scale bars: 50µm. Data in d are mean ± s.d. Electronic supplementary material 7 (PDF 518 kb)

Supplementary movie 1. Secondary sprouting in a wild-type embryo. Movie corresponding to the still images shown in Fig. 6a-a’’’ depicting venous sprouting in a *flt4:mCitrine;* *flt1:tdTomato* transgenic embryo. Electronic supplementary material 8 (AVI 4232 kb)

Supplementary movie 2. In *grb2b*^*mu404*^ mutants, secondary sprouting is defective. Aberrant venous sprouting in a *flt4:mCitrine;* *flt1:tdTomato* transgenic *grb2b*^*mu404*^ mutant embryo. The movie corresponds to the still images shown in Fig. 6c-c’’’. Electronic supplementary material 9 (AVI 4083 kb)

Supplementary movie 3. Defective venous sprouting in a *grb2b*^*mu404*^ mutant embryo. Movie corresponding to the still images in Fig. 6e-e’’’ showing the formation of an intersegmental vein in a *flt4:mCitrine;* *flt1:tdTomato* transgenic *grb2b*^*mu404*^ mutant. Electronic supplementary material 10 (AVI 5971 kb)

## Data Availability

Not applicable.
