# Supplementary figures and images for: The adaptor protein Grb2b is an essential modulator for lympho-venous sprout formation in the zebrafish trunk
Source: Angiogenesis. 2021 Mar 7;24(2):345–62. doi: 10.1007/s10456-021-09774-w (PMC8205915; doi:10.1007/s10456-021-09774-w)

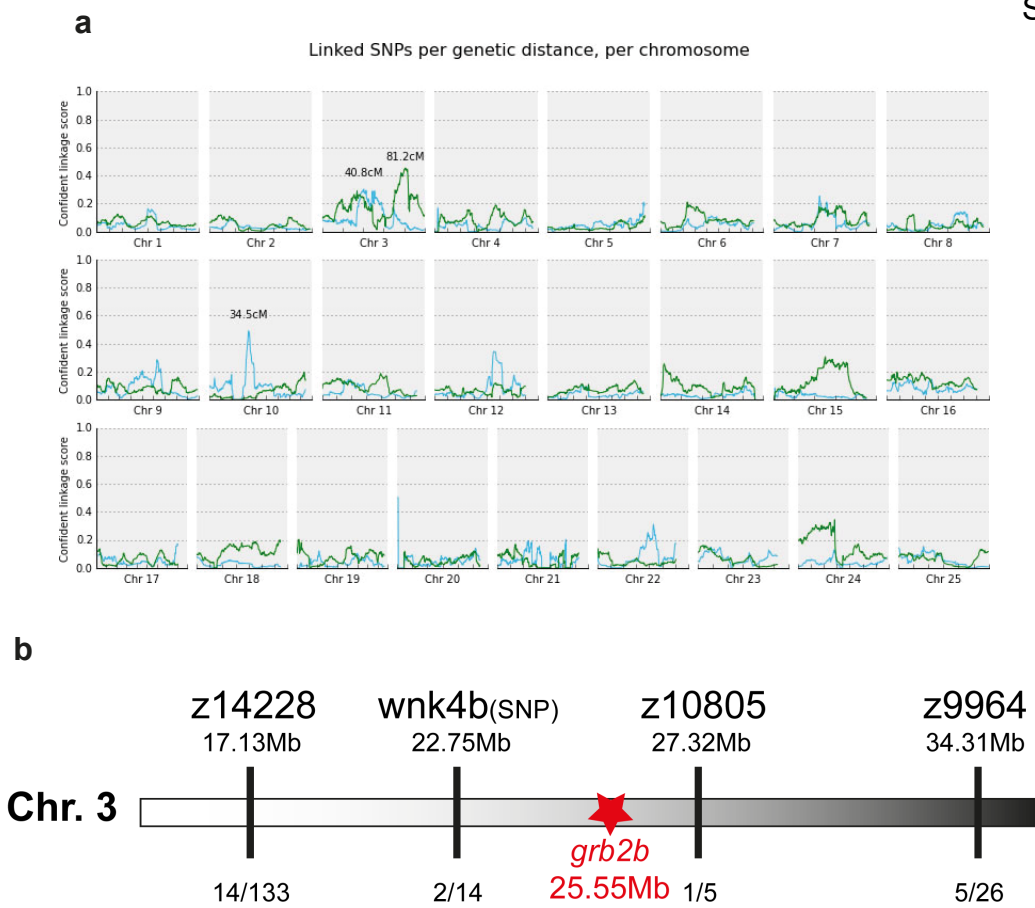

Supplement: Supplementary file 1 — Supplementary Figure 1. tabula rasa links to a genomic interval on Chromosome 3. (a) Linkage analysis of each chromosome for the tabula rasa mutation based on next-generation sequencing data from pooled mutant and sibling DNA. Homozygosity mapping taking either all identified SNPs into account (green lines) or employing only SNPs that appeared to result from the ENU treatment of the screen fish (blue lines) indicated a possible linkage of the tabula rasa mutation to chromosome 3. (b) By conventional genetic linkage analysis using polymorphic markers, linkage to chromosome 3 was verified and the mutation was mapped to a region between an informative SNP in the wnk4b gene and the marker z10805 (position 22.75Mb and 27.32Mb according to the genome assembly Zv9). Candidate mutations in the region were subsequently identified employing the next-generation sequencing data. Electronic supplementary material 1 (PDF 387 kb) [file 10456_2021_9774_MOESM1_ESM.pdf]

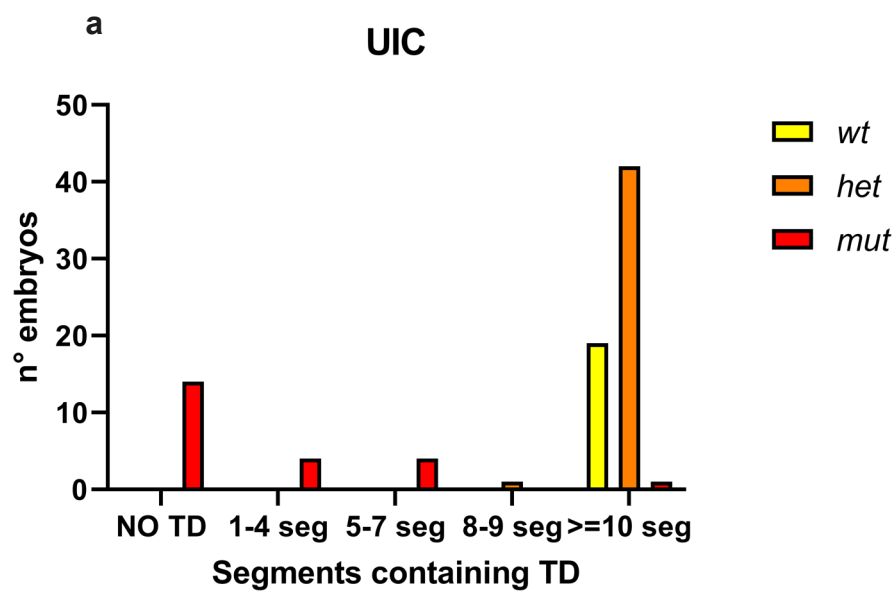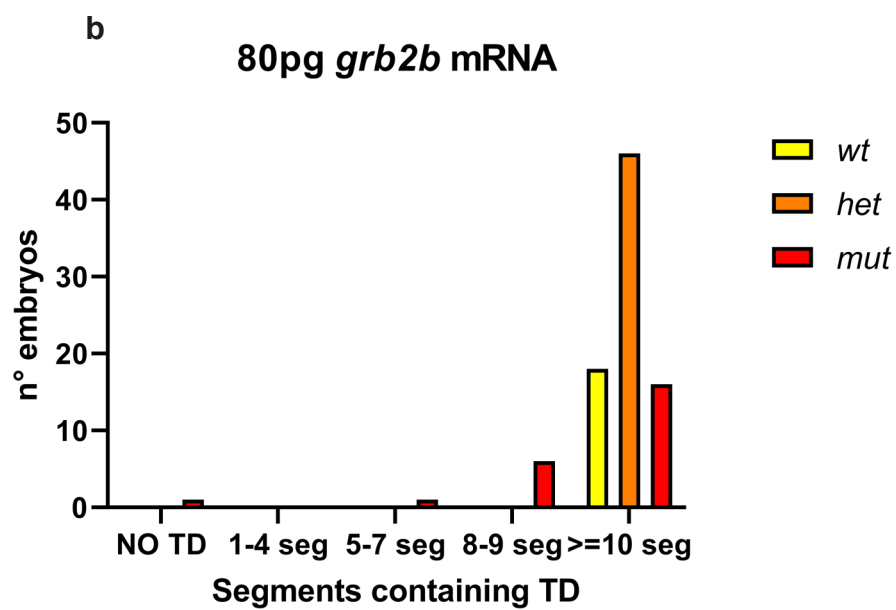

Supplement: Supplementary file 2 — Supplementary Figure 2. The tabula rasa lymphatic phenotype is rescued by grb2b mRNA injections. (a) TD quantification in 10 trunk segments of embryos from a tabula rasa in-cross as un-injected control (UIC). Most of the mutants do not develop a TD or they only have few TD fragments. wt: n=19, het: n=43, mut: n=23. (b) Embryos from a tabula rasa in-cross injected with 80pg of grb2b mRNA showing a rescue of the TD defects in mutant embryos. wt: n=18, het: n=46, mut: n=24. TD: thoracic duct. Electronic supplementary material 2 (PDF 171 kb) [file 10456_2021_9774_MOESM2_ESM.pdf]

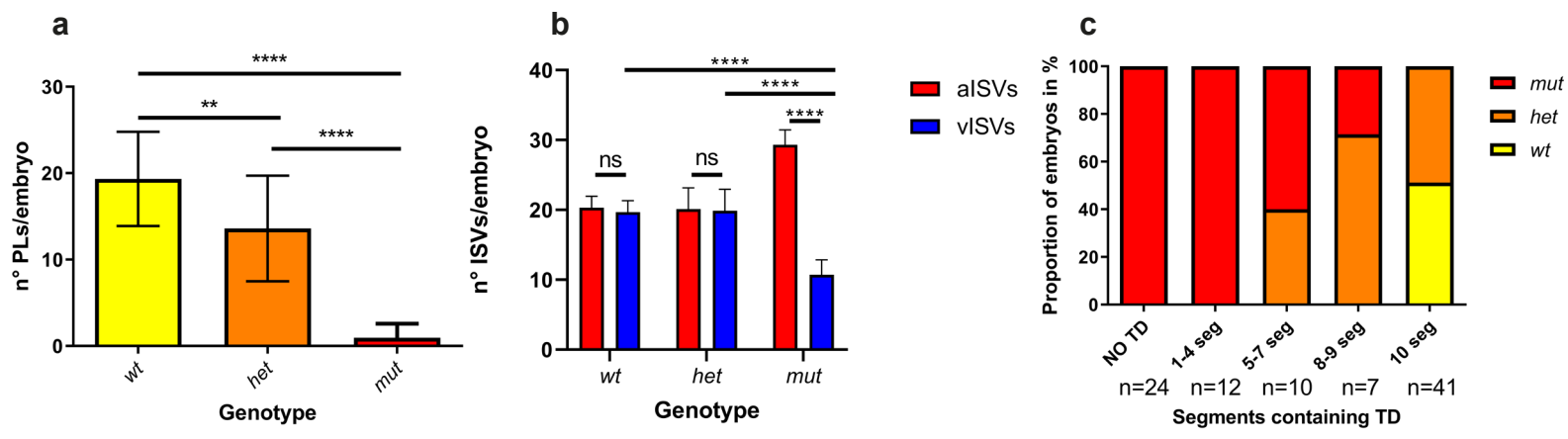

Supplement: Supplementary file 3 — Supplementary Figure 3. The grb2bmu404 allele causes defects that are comparable to the tabula rasa allele. (a) PL cells are significantly decreased in grb2bmu404 mutants compared to wild types and heterozygotes at 48hpf. wt: n= 12; het: n= 51; mut: n= 25. ** Between wt and het: P value 0.0033 (Mann–Whitney). ****Between wt and mut: P value <0.0001 (Mann–Whitney). **** Between het an mut: P value <0.0001 (Mann–Whitney). (b) In grb2bmu404 mutants, the number of vISVs is significantly reduced compared to aISVs. The total number of quantified ISVs per embryo was 40. wt: n= 6; het: n= 27; mut: n= 10. **** Between aISVs mut and vISVs mut: P value <0.0001 (t test, two-tailed). **** Between vISVs wt and vISVs mut: P value <0.0001 (t test, two-tailed). **** Between vISVs het and vISVs mut: P value <0.0001 (Mann–Whitney). (c) TD fragments are missing in grb2bmu404 mutant embryos at 5dpf, as shown in the quantification. Segments containing TD were quantified over the length of 10 somites. PL: parachordal lymphangioblast, TD: thoracic duct, aISV: arterial intersegmental vessel, vISV: venous intersegmental vessel; ns: not significant. Data in a, b are mean ± s.d. Electronic supplementary material 3 (PDF 160 kb) [file 10456_2021_9774_MOESM3_ESM.pdf]

*grb2b* mRNA 32hpf

*grb2a* mRNA 32hpf

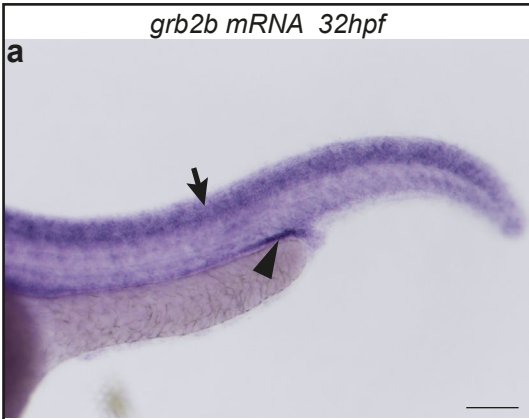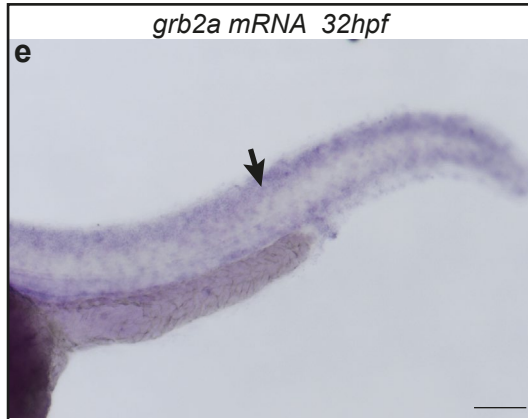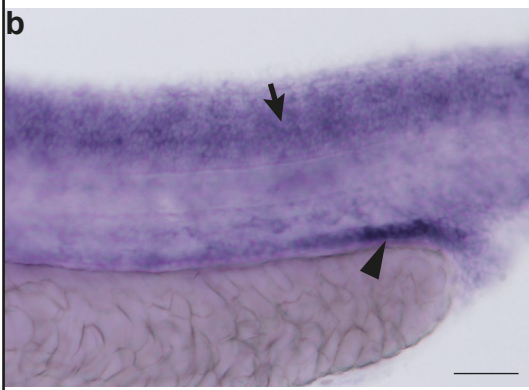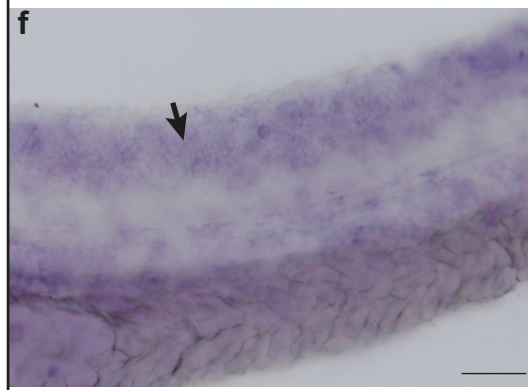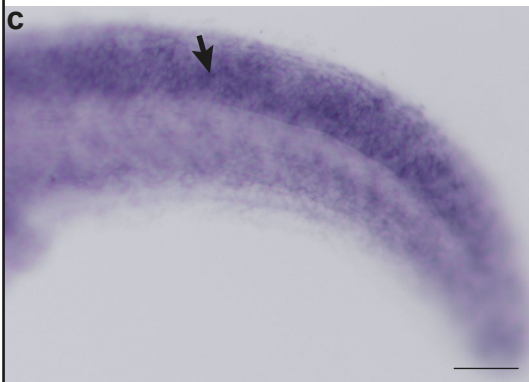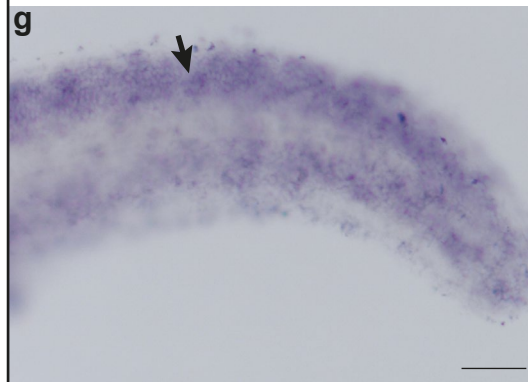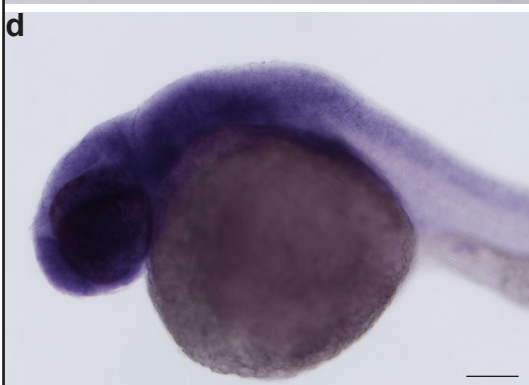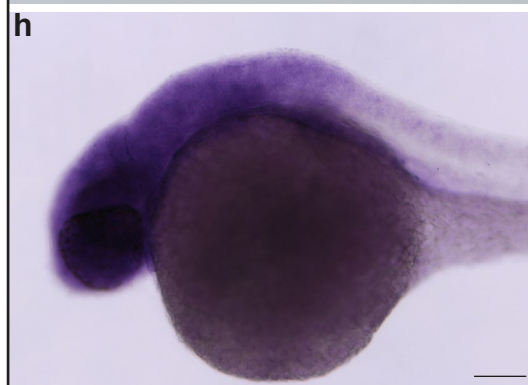

Supplement: Supplementary file 4 — Supplementary Figure 4. Expression pattern of grb2b and grb2a mRNA during venous sprouting. (a-g) In situ hybridization against grb2b on wild-type embryos at 32hpf. Within the trunk, grb2b mRNA can be detected in various tissues with the strongest expression being evident within the central nervous system (arrows) and the distal part of the pronephros (arrow head) (a-c). In the head region, grb2b is ubiquitously expressed (d). (e-h) Detection of grb2a mRNA in wild-type embryos at 32hpf. As for grb2b, grb2a transcripts can be detected in various different tissues within the trunk (e-g) and head region (h) with a prominent expression within the spinal cord (arrows). Scale bars in a, g, e, h: 100µm; in b, c and f, g: 50µm. Electronic supplementary material 4 (PDF 448 kb) [file 10456_2021_9774_MOESM4_ESM.pdf]

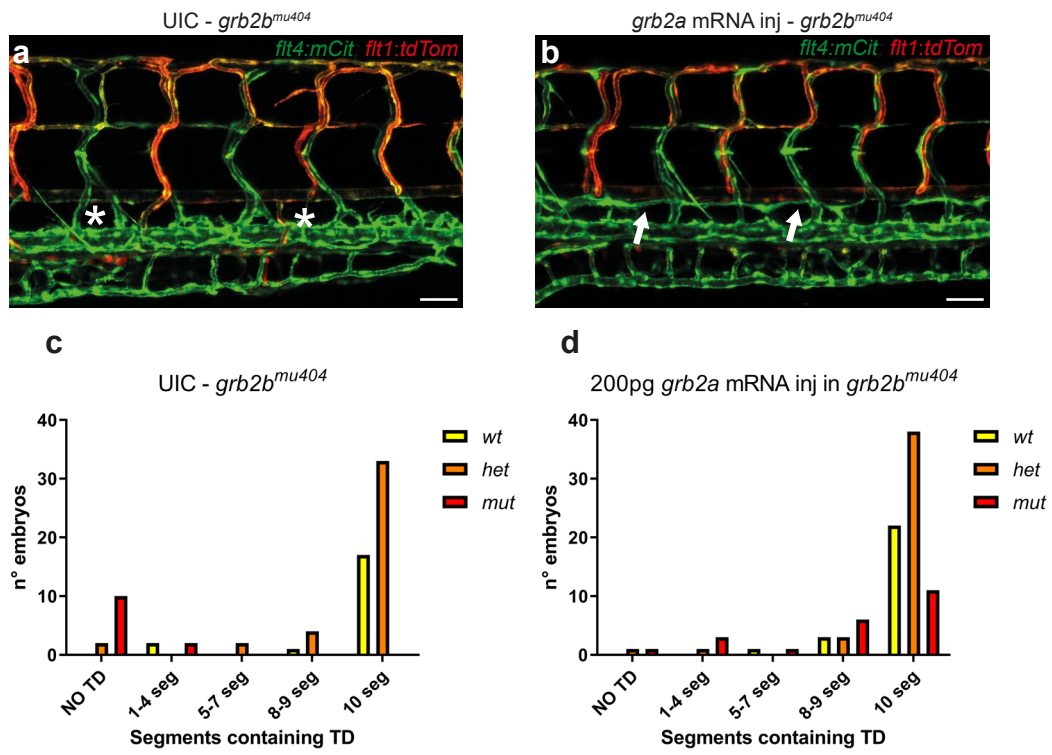

Supplement: Supplementary file 5 — Supplementary Figure 5. grb2a can compensate for the loss of grb2b function in trunk lymphatics. (a,b) Confocal projections of grb2bmu404 mutant embryos as un-injected control (UIC, a) and injected with grb2a mRNA (b). flt4:mCitrine is shown in green, flt1:tdTomato in red. Note the absence of TD in UIC embryos (asterisk) and the presence of TD upon grb2a mRNA injection (arrows). (c,d) Quantification of TD formation in embryos from a grb2bmu404 in-cross that were not injected (c) (wt: n=20, het: n=41, mut: n=12) or that were injected with 200pg of grb2a mRNA (d) (wt: n=26, het: n=43, mut: n=22) indicates that Grb2a can rescue the grb2b mutant TD phenotype. TD: thoracic duct. Scale bars: 50µm. Electronic supplementary material 5 (PDF 333 kb) [file 10456_2021_9774_MOESM5_ESM.pdf]

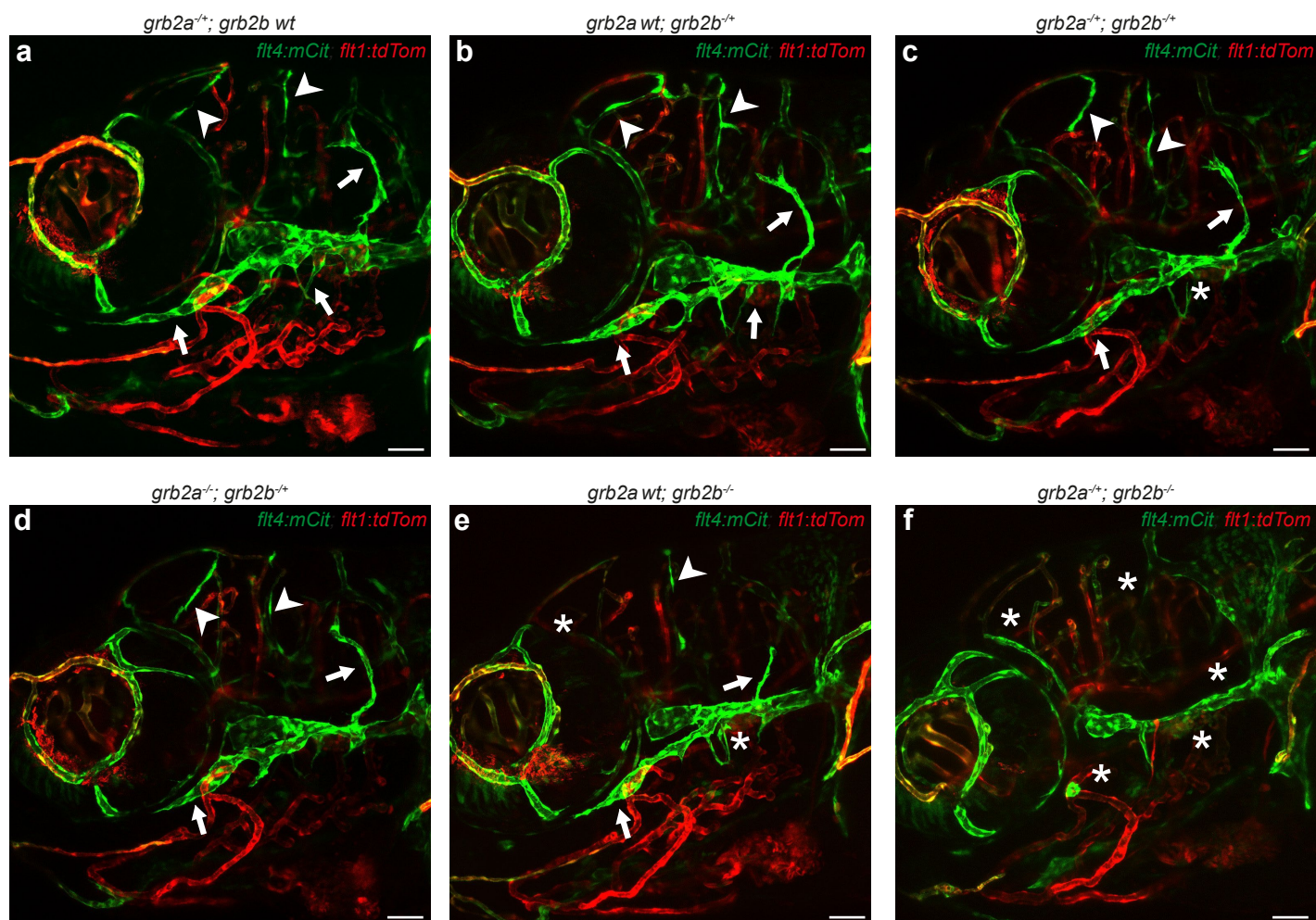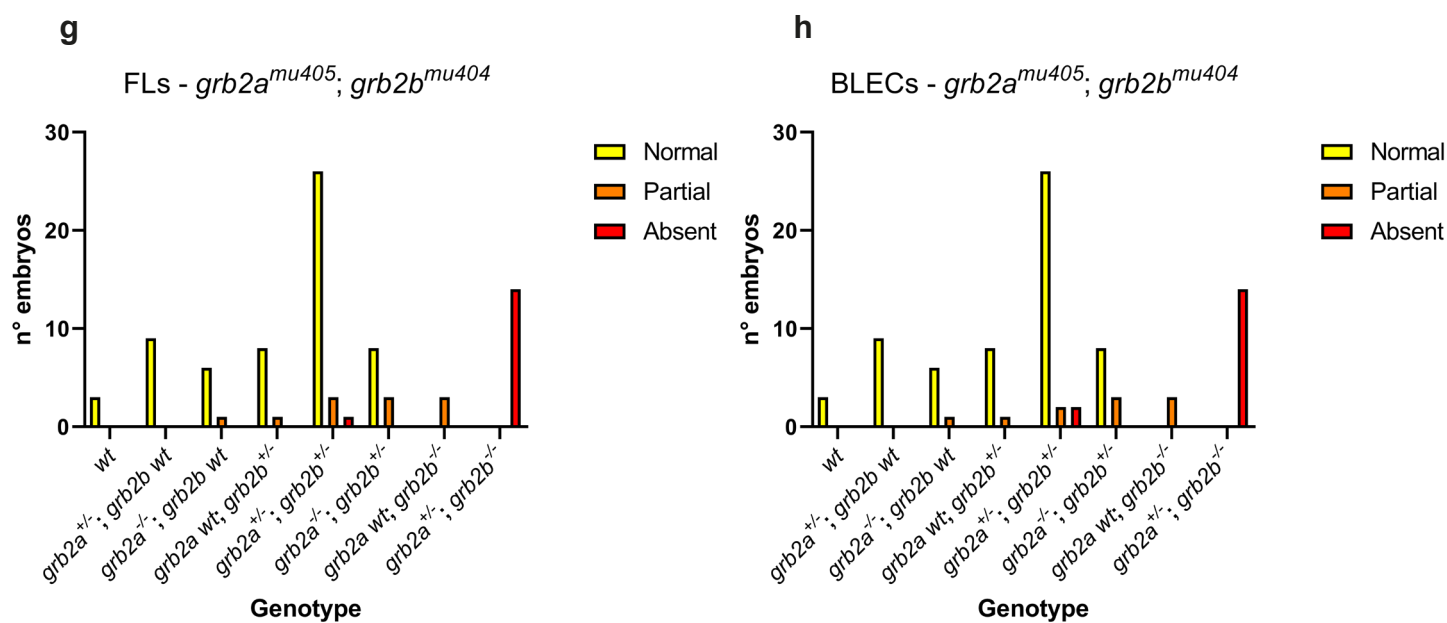

Supplement: Supplementary file 6 — Supplementary Figure 6. grb2a and grb2b are required for the development of the facial lymphatic system and of brain lymphatic endothelial cells. (a-f) Confocal projections of the zebrafish head at 4dpf in embryos with the indicated genotypes. Veins and lymphatics are shown in green (flt4:mCitrine) and arterial blood vessels in red (flt1:tdTomato). Arrows point at the different structures of FLs and arrowheads highlight BLECs. Asterisks mark the absence of those structures. (g,h) Quantification of FLs and BLECs structures in a grb2amu405; grb2bmu404 double heterozygous in-cross at 4dpf. The phenotypes have been divided into three categories: normal, when all structures are present; partial, when some structures are not developed; absent, when the respective structures are missing completely. Note that within the same embryo, BLECs and FLs were mostly affected to a similar extent. Double homozygous embryos were not incorporated into the analysis since they either did not survive until 4dpf or they showed signs of tissue necrosis. FLs: facial lymphatics, BLECs: brain lymphatic endothelial cells. Scale bars: 50µm. Electronic supplementary material 6 (PDF 1036 kb) [file 10456_2021_9774_MOESM6_ESM.pdf]

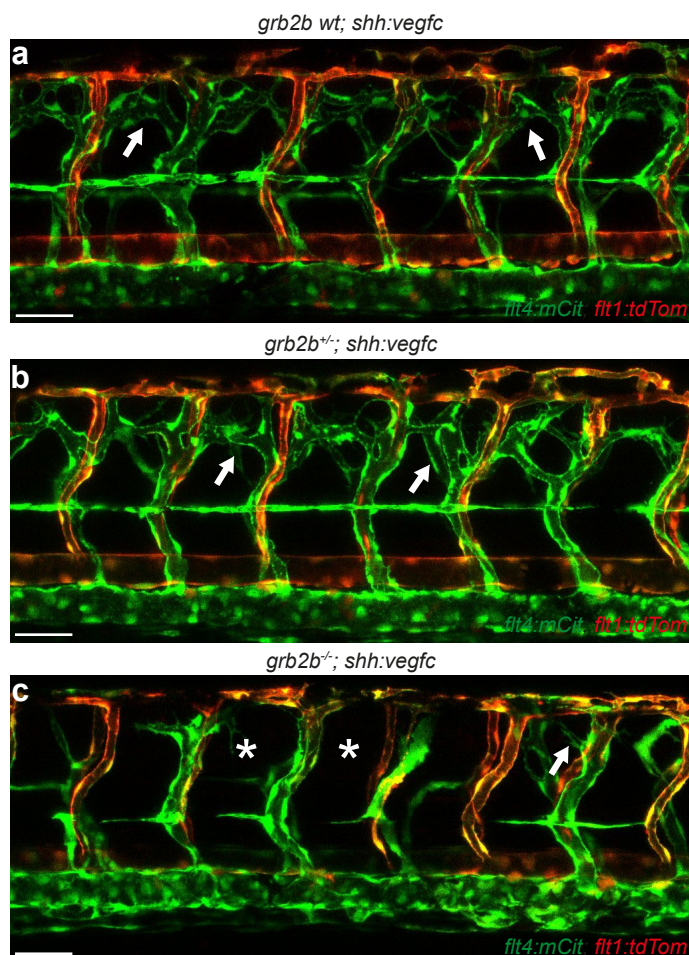

**d**

*grb2b*<sup>*mu404*</sup>; *shh:vegfc-IRES-mTurquoise*

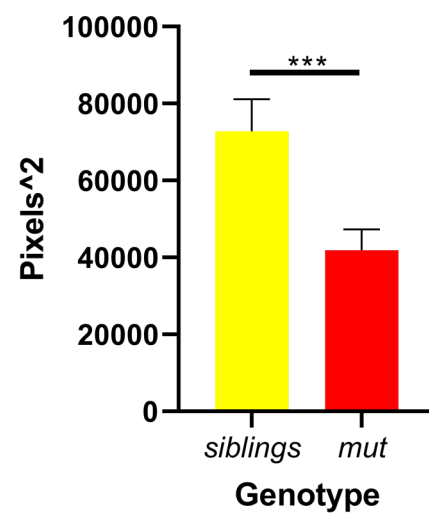

Supplement: Supplementary file 7 — Supplementary Figure 7. Grb2b acts downstream of the Vegfc/Vegfr3 pathway. (a-c) Confocal projections of shh:vegfc-IRES-mTurquoise; grb2bmu404 wild-type, heterozygous and homozygous embryos. The over-expression of Vegfc in the floorplate causes a dominant hyper-branching of ISVs (arrows in a, b), which is strongly suppressed by a complete loss of grb2b (marked by asterisk in c). (d) Quantification of the total vessel area in the dorsal aspect of ISVs in grb2bmu404 mutants or siblings expressing the shh:vegfc-IRES-mTurquoise transgene, showing a significant decrease of venous hyper-sprouting in embryos lacking both functional copies of grb2b. Siblings: n= 5; mut: n= 9. *** Between siblings and mut: P value =0.001 (Mann–Whitney). Scale bars: 50µm. Data in d are mean ± s.d. Electronic supplementary material 7 (PDF 518 kb) [file 10456_2021_9774_MOESM7_ESM.pdf]
